# Genomic Structure of and Genome-Wide Recombination in the *Saccharomyces cerevisiae* S288C Progenitor Isolate EM93

**DOI:** 10.1371/journal.pone.0025211

**Published:** 2011-09-26

**Authors:** Anders Esberg, Ludo A. H. Muller, John H. McCusker

**Affiliations:** Department of Molecular Genetics and Microbiology, Duke University Medical Center, Durham, North Carolina, United States of America; National Cancer Institute, United States of America

## Abstract

The diploid isolate EM93 is the main ancestor to the widely used *Saccharomyces cerevisiae* haploid laboratory strain, S288C. In this study, we generate a high-resolution overview of the genetic differences between EM93 and S288C. We show that EM93 is heterozygous for >45,000 polymorphisms, including large sequence polymorphisms, such as deletions and a *Saccharomyces paradoxus* introgression. We also find that many large sequence polymorphisms (LSPs) are associated with Ty-elements and sub-telomeric regions. We identified 2,965 genetic markers, which we then used to genotype 120 EM93 tetrads. In addition to deducing the structures of all EM93 chromosomes, we estimate that the average EM93 meiosis produces 144 detectable recombination events, consisting of 87 crossover and 31 non-crossover gene conversion events. Of the 50 polymorphisms showing the highest levels of non-crossover gene conversions, only three deviated from parity, all of which were near heterozygous LSPs. We find that non-telomeric heterozygous LSPs significantly reduce meiotic recombination in adjacent intervals, while sub-telomeric LSPs have no discernable effect on recombination. We identified 203 recombination hotspots, relatively few of which are hot for both non-crossover gene conversions and crossovers. Strikingly, we find that recombination hotspots show limited conservation. Some novel hotspots are found adjacent to heterozygous LSPs that eliminate other hotspots, suggesting that hotspots may appear and disappear relatively rapidly.

## Introduction

Meiosis is the specialized cell division by which the diploid cells of sexually reproducing organisms undergo a single round of DNA replication followed by two successive cell divisions to generate haploid gametes. Meiotic recombination is of great importance because it promotes genetic diversity by creating new and potentially beneficial genetic combinations, purging harmful mutations [1], [2], and ensuring interhomolog chromosomal association important for proper chromosomal segregation [3], [4]. In most organisms, recombination events are distributed non-randomly throughout the genome [5], which gives rise to dispersed cold and hot regions [6].

Much of our understanding of meiotic recombination has been obtained from two complementary types of genome-wide studies of meiotic recombination in *Saccharomyces cerevisiae*. One type of study, which utilizes strains that lack heterozygosities, determines the locations of recombination initiating double strand breaks (DSBs) [5], [7], [8], [9]. The second type of study, which utilizes strains with multiple heterozygosities that may affect recombination, determines the segregation of multiple heterozygous markers, identifying reciprocal and gene conversion recombination events, as well as assessing interference [10], [11].

In this work, we perform a genome-wide study of meiotic recombination in, and determine the genome structure of, EM93, a natural isolate that is the main ancestor to the most frequently used *S. cerevisiae* laboratory strain, S288C [12]. First, we compare the genome of EM93, which is a heterothallic, multiply heterozygous wild-type isolate, to that of S288C by hybridizing DNA from eight EM93 meiotic segregants to S288C-based GENECHIP *S. cerevisiae* Tiling 1.0R Arrays (Affymetrix). We use this Tiling Array data to obtain a high-resolution overview of genomic similarities and differences between the natural isolate EM93 and that of the laboratory strain S288C, which identified multiple polymorphisms, including a heterozygous *S. paradoxus* introgression in EM93. Second, using the hybridization profiles of the individual probes present on the Tiling Array, we identified heterozygous genetic markers that were used to design an EM93 genotyping array. Utilizing the genotyping array, we genotyped 480 segregants (from 120 EM93 tetrads) for 2,965 heterozygous genetic markers and assembled a deduced EM93 genomic structure. Finally, we determined the frequency and distributions and frequencies of meiotic reciprocal and non-reciprocal recombination events, as well as gene conversion parity; we find that large sequence polymorphisms have chromosome position-dependent effects on recombination; and we identify both multi-strain and strain-specific recombination hotspots.

## Results

### Tiling Array analysis

**Figure 1:**
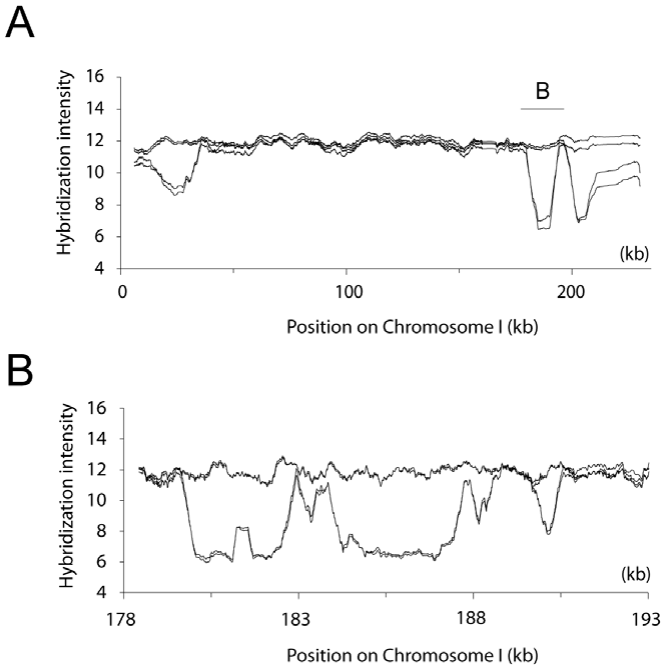
Genomic differences between EM93 and S288C on chromosome I. Labeled DNA from eight segregants originating from two EM93 tetrads was hybridized to the GENECHIP *Saccharomyces cerevisiae* Tiling 1.0R Array. The resulting hybridization profiles allowed us to estimate locations of potential polymorphic regions, deletions, and introgressions present in EM93. (A) Graph shows the hybridization intensities for one tetrad for chromosome I. Polymorphisms between EM93 and S288C are apparent in the hybridization profiles. (B) Close-up of the *Saccharomyces paradoxus* introgression region on chromosome I. The graph shows the region between nucleotide 178,000 and 193,000 (see also Figure S2 and Table S2 LSP2). Graphs were created using a moving average of the hybridization intensities of the probes that have a unique hit in the S288C genome with a window size of 500 probes in A and 200 probes in B.

We hybridized DNA from eight segregants, originating from two EM93 tetrads, to GENECHIP *Saccharomyces cerevisiae* Tiling 1.0R Arrays. The Tiling Array, which carries over 3.2 million S288C perfect-match and mismatch probes with a median probe offset of 4 base pairs [13], provided a high resolution overview of the genomic differences between EM93 and S288C (Figure 1A). EM93 is a heterothallic heterozygous diploid isolate [12] and as the four segregants from each of two EM93 tetrads were hybridized to the Tiling Array, homozygous as well as heterozygous polymorphisms were detected.

Consistent with the 88% of the S288C genome predicted to originate from EM93 [12], approximately 77% of the EM93 genome is heterozygous (based on the S288C genome size and on percent coverage from first to last heterozygous marker for each chromosome). As described below, regions lacking genetic markers were usually fairly large (e.g. 785 kB on Chromosome IV). Based on the Tiling Array hybridization profiles, the regions lacking genetic markers contain predominantly homozygous S288C sequence, with occasional homozygous non-S288C sequences (Table S2, LSP14, and Figure S3F).

### A heterozygous *Saccharomyces paradoxus* introgression on chromosome I

We found that the same region shown to be an *S. paradoxus* introgression in the clinically derived strain YJM789 [14] shows 2∶2 segregation of hybridization intensity on the Tiling Array (Figure 1). To determine if EM93 carries an *S. paradoxus* introgression similar to that identified in YJM789, we designed primer pairs that would amplify either an S288C-like or an YJM789/*S. paradoxus*-like sequence. By PCR and sequencing, we confirmed that a 3.9 kB *S. paradoxus* introgression similar to that present in YJM789 is present on one copy of chromosome I in EM93 (Figure S2).

### Sub-telomeric Large Sequence Polymorphisms

Of the thirty-two sub-telomeric regions (i.e. within 20 kb of the corresponding S288C telomere), the Tiling Array hybridization profiles identified twelve that displayed reduced hybridization intensity, consistent with large sequence polymorphisms (LSPs) relative to S288C. Eleven of these sub-telomeric LSPs were heterozygous (Table S2 and Figure S3). However one sub-telomeric LSP (#14), located on chromosome VII between nucleotide 1,069,041 and 1,076,119, was predicted to be a homozygous polymorphic or deleted region (Figure S3F). By PCR and sequence analysis, we confirmed that EM93 is homozygous for this LSP, which was 99% identical to the corresponding region in YJM789; thus, EM93 and YJM789, compared to S288C, both carry a polymorphic version of this region.

### LSPs associated with Ty-elements

**Figure 2:**
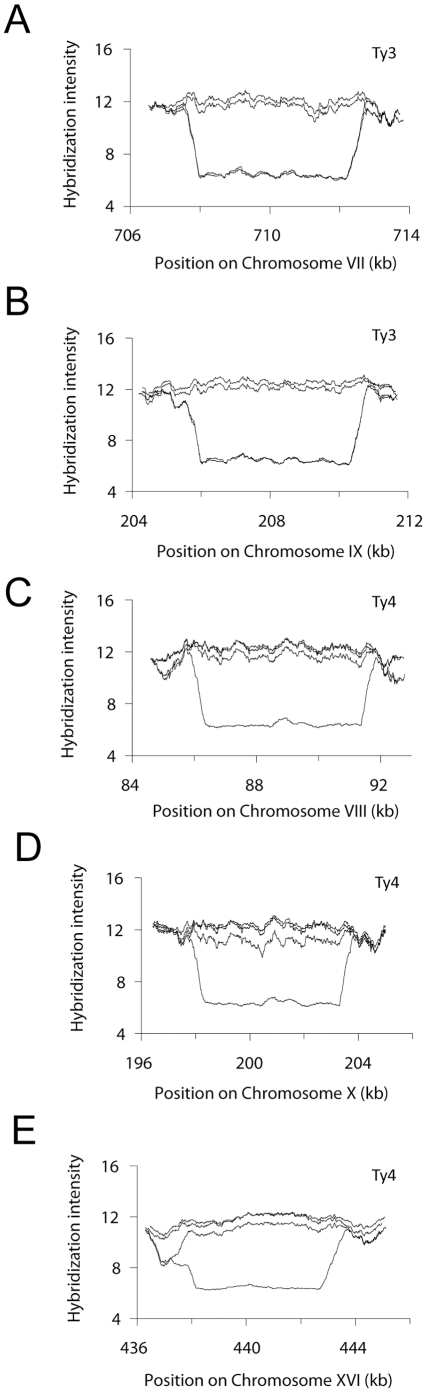
Ty-associated polymorphic regions. Graphs show hybridization intensities for four segregants originating from one EM93 tetrad. (A–B) EM93 lacks Ty3 on chromosome VII and is heterozygous for Ty3 on chromosome IX (Table S2: LSP13 and 17). (C–E) EM93 is heterozygous for all three Ty4-elements located on chromosomes VII, X, and XVI (Table S2: LSP15, 22, and 27, respectively). Graphs are created using a moving average of the hybridization intensities with a moving window size of 200 probes.

Based on the hybridization profiles and estimated coordinates of the 15 non-sub-telomeric LSPs in EM93, 12 LSPs corresponded to the locations of Ty-elements or long terminal repeats (LTRs) in S288C (Table S2). Upon closer examination of Ty-associated polymorphic regions, we observed that the hybridization profile of probes specific to Ty3 displayed 2∶2 segregation in both of the hybridized tetrads (Figure 2A–B, Table S2 LSP13 and 17), which was surprising since S288C carries two copies of Ty3. By PCR and sequencing analysis, we found that EM93 lacks Ty3 on chromosome VII and is heterozygous for Ty3 on chromosome IX (Figure 2A–B, Figure S4), thus explaining the observed 2∶2 segregation. We also observed that one out of the eight hybridized segregants did not hybridize to Ty4-specific probes (Figure 2C–E, Table S2 LSP 15, 22, and 27), which, again, was surprising since S288C has 3 copies of Ty4, located on chromosome VIII, X, and XVI. By PCR analysis, we found that EM93 is heterozygous for all three Ty4-elements, thus explaining the hybridization pattern observed on the Tiling Array for Ty4 (Figure S5). (Array hybridization data for other, mostly Ty- and LTR-associated LSPs are shown in Figure S6.) Therefore, at least five of the 12 Ty-associated LSPs are, in fact, due to the presence/absence of Ty-elements in EM93.

### Locations and configurations of heterozygous markers in EM93 chromosomes

In addition to identifying LSPs, the hybridization profiles of the probes present on the Tiling Array identified >45,000 polymorphisms. We used these many heterozygous polymorphisms as genetic markers to design a custom 8×15K Agilent genotyping array carrying 5 copies of each of 2,965 genetic markers (Table S4), with an average inter-marker distance of 3.4 kb covering 77% of the genome, to investigate meiosis and recombination in EM93.

**Figure 3:**
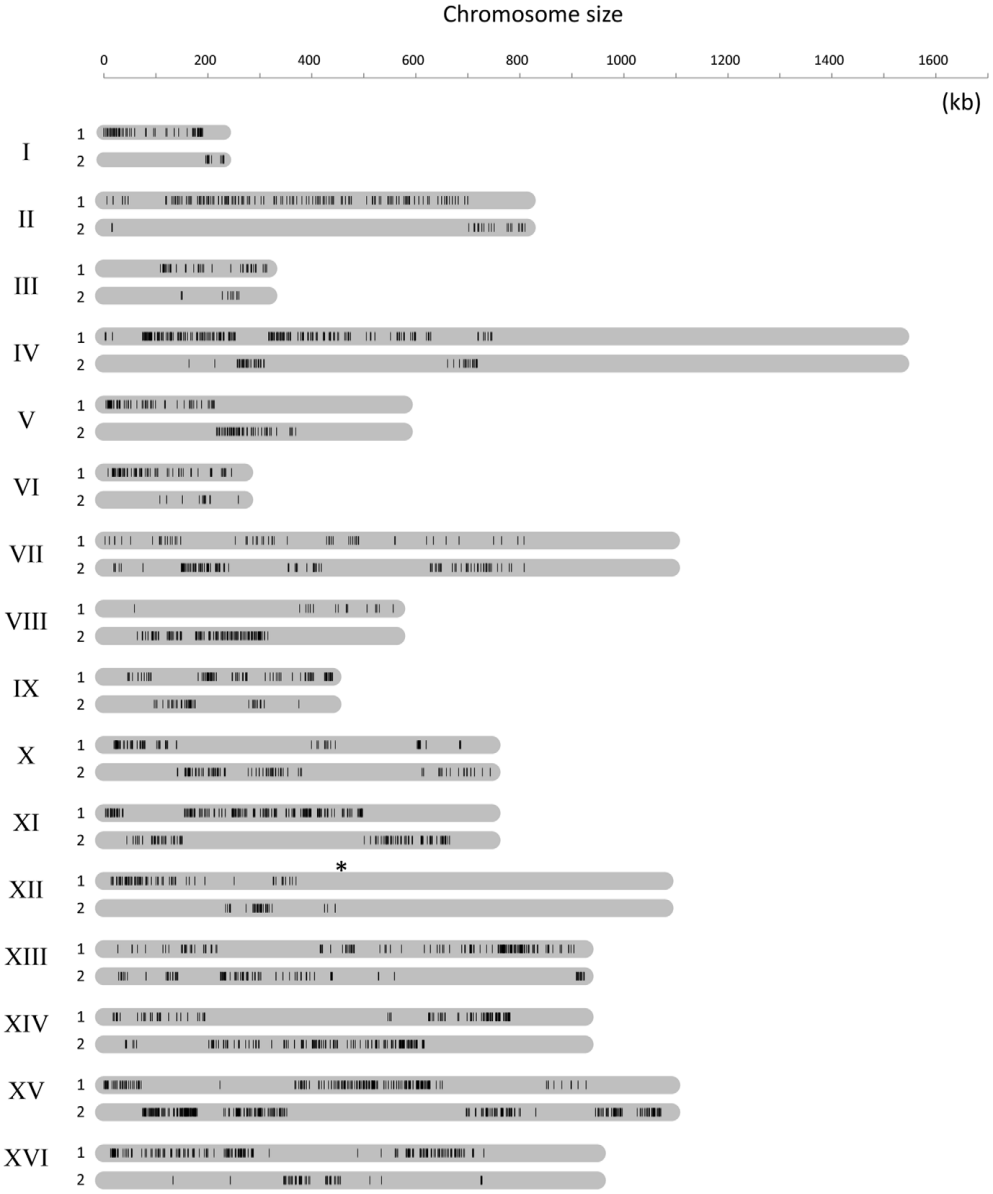
Deduced genomic structure of EM93. The 1.42 million genotyping calls generated by hybridizing 480 segregants (120 tetrads) to custom Agilent genotyping arrays were used to determine the order and organization of the two heterozygous marker-types, S288C-like or non-S288C. Across each chromosome, the nearest marker-type was determined by linkage analysis. This provides a deduced genomic organization of EM93 and with information regarding the size and distribution of S288C-like and non-S288C regions across the EM93 genome. To clarify and to enhance the genomic overview, only the non-S288C marker type (black-bars) for both homologous chromosomes (1 and 2) is shown. Position of the rDNA is indicated with a (*). Regions without markers are homozygous, based on Tiling Array hybridization, and are mostly, if not entirely, S288C-like.

For genotyping, labeled DNA from 480 segregants (4 segregants ×120 tetrads) was separately hybridized to the custom genotyping arrays (see Materials and Methods). Based on the hybridization intensity, a genotype call was made for each marker in every segregant (an average hybridization intensity difference of 30.5 fold and *p-value <0.05* (*student's t-test*) when comparing intensity from S288C-like with non-S288C probes, see Materials and Methods, Figure S1). We then used linkage analysis of the 1.42 million genotype calls to determine the nearest marker-type allowing us to assemble a deduced genomic structure of EM93 (Figure 3). The deduced EM93 genomic structure shows the sizes, locations and configurations of those regions heterozygous for S288C-like and non-S288C markers. In summary, all chromosomes had large regions homozygous for the same marker-type, almost entirely S288C-like, as well as heterozygous regions with interspersed S288C-like and non-S288C markers; all chromosomes showed evidence of heterozygosity compared to S288C.

### Recombination in EM93

**Figure 4:**
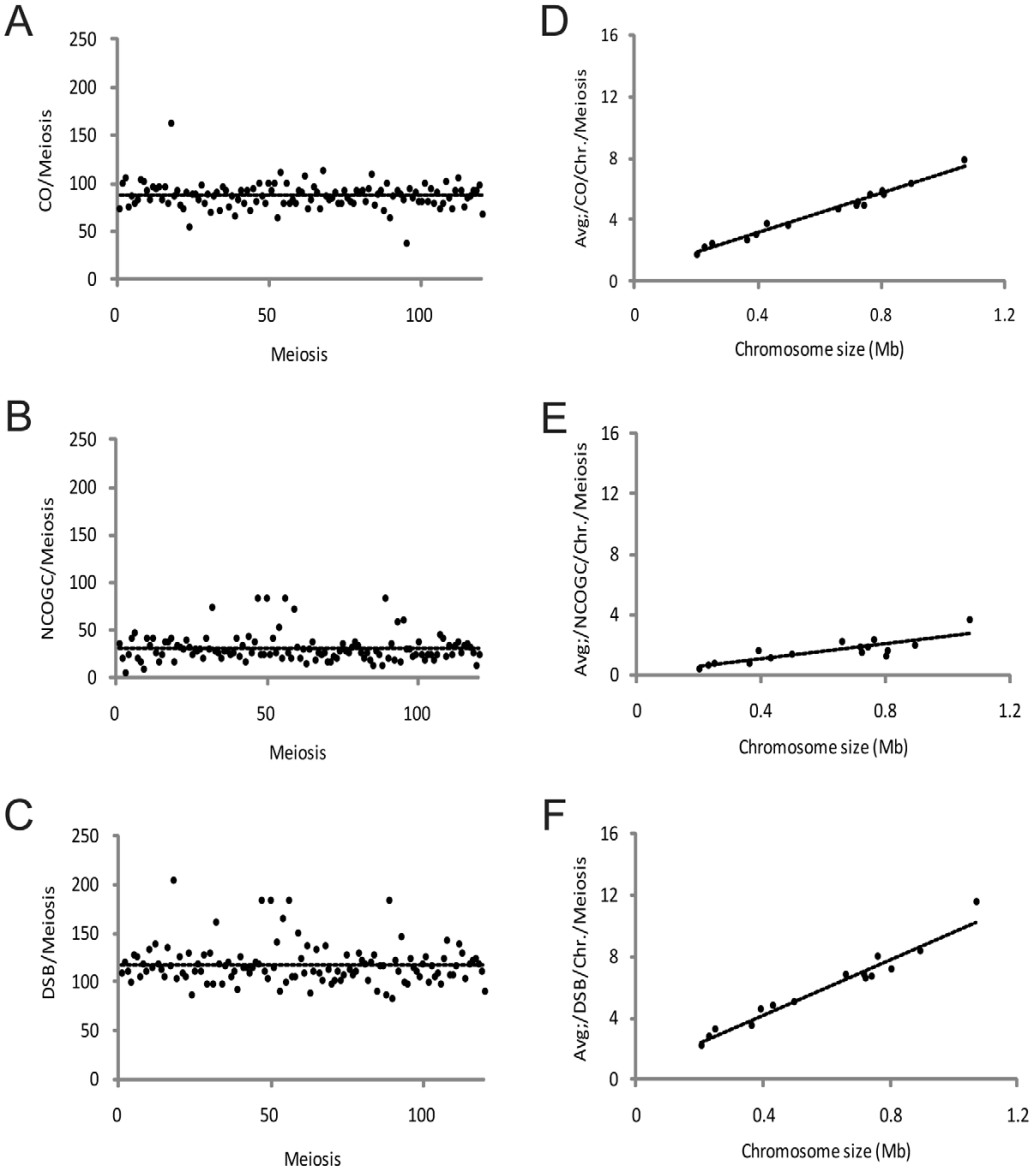
Recombination outcomes in EM93. The total number of crossovers (CO) (A) and non-crossover gene conversion (NCOGC) (B) events was identified in 120 tetrads. As both CO and NCOGC events are induced by a double-stranded-break (DSB), the sum of CO and NCOGC events reflects the total number of DSBs induced per genome and meiosis (C). The number of COs (D), NCOGCs (E), and DSBs (F) events per chromosome is linearly related to the chromosomal length. This corresponds to 0.6 obligatory CO, plus an additional 6.5 COs per megabases (Mb); 0.04 obligatory NCOGC, plus an extra 2.6 NCOGCs per Mb; 0.6 obligatory DSB plus an additional 9.0 DSBs per Mb.

To analyze recombination events in EM93, the genotype calls for each marker were grouped by tetrad and recombination events were designated as gene conversions (GCs), or as reciprocal crossovers (COs) (Materials and Methods). In total, we detected 8,501 COs and 4,240 GCs in 120 tetrads. As the genotyping array lacks heterozygous probes in 23% of the genome, we adjusted for undetectable COs, giving a total of 10,456 COs in 120 tetrads (8,501 COs ×1.23). Of the 4,240 identified GCs, 1,264 were crossover associated gene conversions (COAGCs) and 2,976 were non-crossover associated gene conversions (NCOGCs). Adjusted for genome coverage (2,967×1.23), we estimate that there are 31 NCOGCs per EM93 meiosis. However, given the average marker spacing of 3.4 kB, many NCOGCs, as well as COAGCs, occur between markers and are not detected; thus, the 31 NCOGCs per EM93 meiosis is a minimum estimate. In summary, our data suggests that EM93 generates a minimum of 118 DSBs per meiosis that, upon repair, give rise to an average of 87 COs and 31 NCOGCs per meiosis (Figure 4A–C).

The number of DSBs, COs, and NCOGCs per chromosome is linearly related to the physical chromosomal length (in this analysis, the rDNA on chromosome XII is excluded) with intercepts of 0.6 and 0.04 [10] for CO and NCOGC, respectively, corresponding to obligatory events per chromosome. We calculate that there are 6.5 COs per megabase (Mb) and 2.6 NCOGCs per Mb, which correspond to 9.0 DSBs per Mb (Figure 4D–F). Of the 1,920 chromosomes analyzed, only five had no detectable recombination events (chromosome I (n = 2) and one each for chromosomes III, V and IX); this equals 0.26% of the chromosomes investigated. Nevertheless, considering the likely number of undetectable events due to homozygous regions that lack genetic markers, our data are consistent with the hypothesis of an obligate chiasma per chromosome pair per meiosis [15].

### Crossover interference

To investigate CO interference in EM93, we selected chromosomes XI and XIV that had heterozygous genetic markers across the whole chromosomes. Briefly, as has been described previously [16], chromosomes XI and XV were divided into approximately 50 kb intervals and interference was determined by comparing the distribution of CO in the adjacent interval in the same tetrad for the case where the reference interval contains a CO or not. We observed CO interference in 6 of 32 intervals tested (Fisher-exact test corrected for multiple comparisons (*p-value* = *0.0025* for chromosome XI *and 0.0042* for chromosome XV) (Table S5 and Table S6). However, clear evidence of CO interference was observed for both of the chromosomes when analyzing the complete data for the entire chromosome, both chromosome XI and XV had a *P<0.0001* using Fisher-exact test. This provides strong evidence for CO interference and also suggests that CO interference is not distributed evenly across a chromosome.

### Recombination coldspots and hotspots

To investigate the distribution of meiotic recombination events across the EM93 genome, NCOGC events and CO events between adjacent markers were counted and adjusted to interval size (see Materials and Methods). In general, the regions around centromeres were CO cold-spots, which may serve as a safeguard against loss of centromeric cohesion [17], [18]. Within the 22.9 kB adjacent to centromeres, we observed 0.69 CO/Mb, compared to the genome wide average of 6.5 CO/Mb (Mann-Whitney U test, *p-value <0.001*). Also consistent with centromeres being CO cold-spots, although the closest CO was only 1.8 kb from the centromere on chromosome XI, the average CO distance from the centromere was 5.6

**Figure 5:**
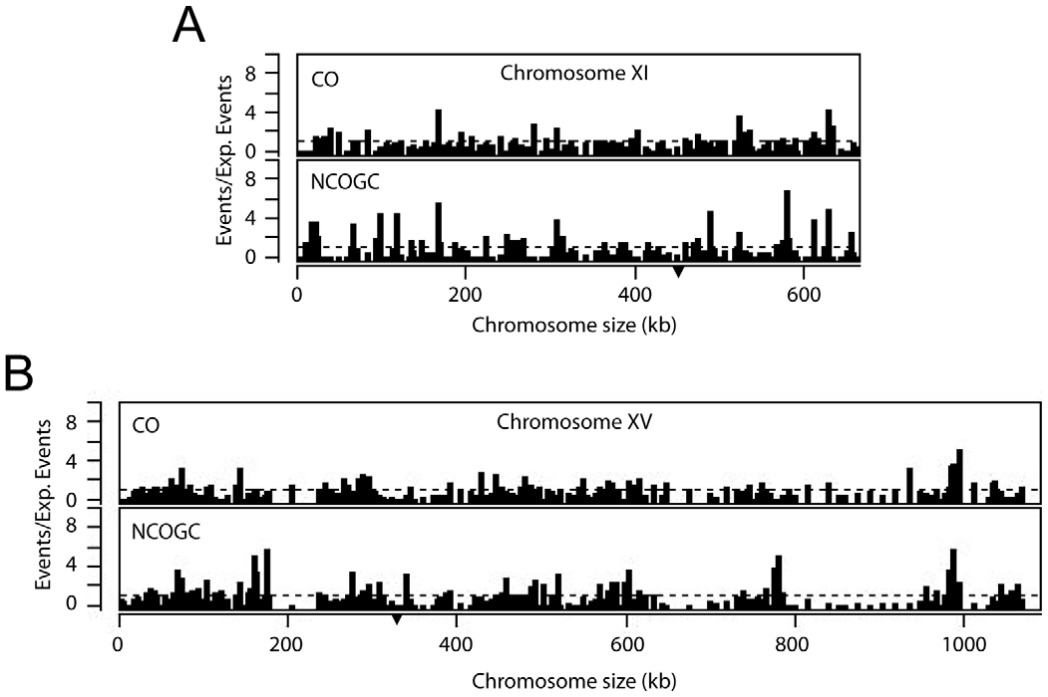
Frequency and distribution of recombination events on chromosome XI and XV. Recombination events across the genome were determined by counting crossover (CO) and non-crossover gene conversion (NCOGC) events between each adjacent marker. The counts were adjusted for the size of the interval. Dividing the number of COs and NCOGCs for each interval by the expected frequency for the respective event (CO∶6.5/Mb/meiosis, NCOGC: 2.6/Mb/Meiosis) generated a recombination score. CO and NCOGC recombination scores are shown for each interval on chromosome XI (A) and chromosome XV (B). Dashed line indicates the expected frequency if considering a homogeneous distribution ( = 1). Position of the centromere is indicated with (▾).

Recombination hotspots were identified as having ≥2-fold more events than expected, based on the genome-wide average of 6.5 COs per Mb per meiosis and 2.6 NCOGCs per Mb per meiosis. We identified 203 recombination hotspots with an average spacing of 45.2 kb (Figure 5, Figure S7, Figure S8, and Table S7). The hottest CO hotspot was on chromosome I between nucleotides 189,875–196,098, where 28.3% of the tetrads had at least one CO. The hottest NCOGC hotspot was on chromosome XVI between nucleotide 726,624 and 730,923, where 17.5% of the tetrads had at least one NCOGC. As suggested by these distinct hottest CO and NCOGC hotspots, and similar to YJM789/S288C [10], most hotspots were hot for only one type of recombination event. As shown in Table S7, intervals that were hot for both COs and NCOGCs were outnumbered by intervals that were hot for only COs that were in turn outnumbered by intervals that were hot for only NCOGCs.

### The effects of heterozygous non-sub-telomeric vs. sub-telomeric LSPs on recombination

To investigate if heterozygous non-sub-telomeric LSPs affect recombination, we compared CO and NCOGC events in these LSP regions to the genome-wide average recombination rate (6.5 per Mb per meiosis for COs and 2.6 per Mb per meiosis for NCOGCs). First, we looked at the regions adjacent to heterozygous non-sub-telomeric LSPs (n = 15) and found that these regions had a 74% and 65% reduction in CO and NCOGC (1.72 CO and 0.79 NCOGC per Mb and meiosis), respectively. Thus, heterozygous non-sub-telomeric LSPs reduce both COs and NCOGCs, and thereby overall recombination. Consistent with these data, no recombination hotspots overlapped with heterozygous non-sub-telomeric LSP regions (Table S2, S7, and S8).

To further evaluate the effect of heterozygous non-sub-telomeric LSPs on recombination, we analyzed the average distance between two hotspots when a heterozygous non-sub-telomeric LSP region is located in-between. Interestingly, we detected a longer average distance between hot-spots, moving from a genome average of 45.2 kb (n = 187) to 69.1 kb (n = 14), for the heterozygous non-sub-telomeric LSP containing regions (Mann-Whitney U test, *p-value* = *0.04*). This suggests that heterozygous non-sub-telomeric LSPs not only reduce the numbers of recombination events but can also lead to hotspot rearrangements.

We then similarly analyzed heterozygous sub-telomeric LSPs. To investigate if heterozygous sub-telomeric LSPs affect recombination, we first analyzed an approximately 30 kb sub-telomeric region for the 23 sub-telomeric regions covered by the Agilent genotyping array. We found that sub-telomeric regions in EM93 had on average 7.7 COs and 1.9 NCOGCs per Mb and meiosis (n = 23), which is similar to the genome-wide average of 6.5 COs and 4.7 NCOGCs per Mb and meiosis. We then compared recombination in the heterozygous LSP sub-telomeric regions (6.1 COs and 2.0 NCOGCs per Mb and meiosis, n = 10) and the LSP-free sub-telomeric regions (9.0 COs and 1.8 NCOGCs per Mb and meiosis, n = 13) and found no significant difference (Mann-Whitney U test, *p-value* = 0.11 and 0.65 for CO and NCOGC, respectively).

Finally, we compared the distance from the ends of the chromosome to the first recombination hotspot between the LSP heterozygous (n = 10) and non-heterozygous (n = 13) sub-telomeric regions. The average distance was similar, 32.8 and 34.6 kb, and no statistical difference (Mann-Whitney U test, *p-valu* = *0.91*) was observed between the LSP heterozygous vs. the non-heterozygous sub-telomeric regions. Therefore, while heterozygous non-sub-telomeric LSPs repress recombination, heterozygous sub-telomeric LSPs have no effect on recombination.

### LSPs and recombination hot and cold spots on chromosomes I and VI

**Figure 6:**
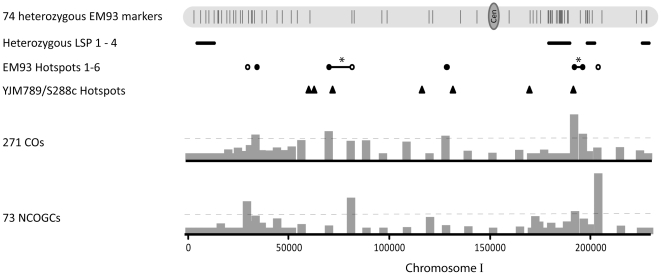
Recombination on Chromosome I. Shown for EM93 chromosome I are the positions of the 74 heterozygous EM93 genetic markers (Table S4); LSPs 1–4 (Table S2); the positions and numbers of recombination events in each interval (bar graphs) and the hotspot threshold (—); and the positions of EM93 crossover (•) and non-crossover gene conversion (○) hotspots (Table S7). * Hotspots found in two or more contiguous intervals are indicated with a solid line in-between the hotspots and are counted as one hotspot (**-**). The positions of YJM789/S288C [10] hotspots are also shown (▴).

Because of their small sizes, multiple LSPs and the markers spaced along their entire lengths, we chose chromosomes I and VI to examine EM93 recombination and to compare recombination in EM93, the primary progenitor of S288C [12], with recombination in the genetically similar YJM789/S288C [10]. The four chromosome I heterozygous LSPs (covering 11.6% of the chromosome) are located between nucleotides 2,828–11,644, 179,666–190,181, 198,866–202,777, and 226,886–230,075 (Table S2, Figure 6). No recombination events were observed between chromosome I nucleotides 407–12,540, 180,009–188,046, 227,257–230,057, and 199,607–202,364. Based on genome-wide recombination, 6.5 CO and 2.6 NCOGC events per Mb would be expected while 0 CO and 0 NCOGC per Mb were observed within and adjacent to these LSPs.

**Figure 7:**
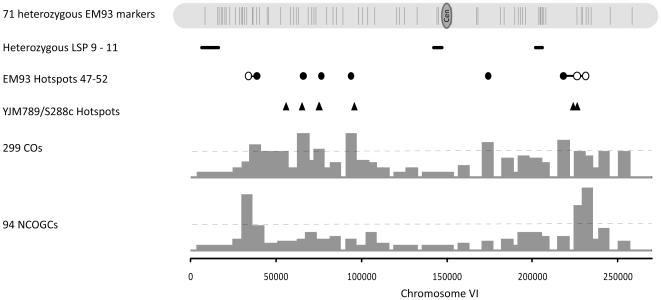
Recombination on Chromosome VI. Shown for EM93 chromosome VI are the positions of the 71 heterozygous EM93 genetic markers (Table S4); LSPs 9–11 (Table S2); the positions and numbers of recombination events in each interval (bar graphs) and the hotspot threshold (—); and the positions of EM93 crossover (•) and non-crossover gene conversion (**Ο**) hotspots (Table S7). * Hotspots found in two or more contiguous intervals are indicated with a solid line in-between the hotspots and are counted as one hotspot (**-**). The positions of YJM789/S288C [10] hotspots are also shown (▴).

Similarly, the three chromosome VI heterozygous LSPs are located between nucleotides 15,320–16,384, 143,952–144,847 and 205,005–205,914 (Table S2, Figure 7). No recombination events were observed between chromosome VI nucleotides 8,181–30,350 and 132,653–152,692; although there was one NCOGC at the distal boundary of LSP11 (205,005–205,914), there was no CO between 203,599–207,830. Based on genome-wide recombination, 6.5 CO and 2.6 NCOGC events per Mb would be expected while 0.1 CO and 0.1 NCOGC per Mb were observed within and adjacent to these three LSPs. Therefore, recombination within and adjacent to these heterozygous LSPs was drastically reduced. In addition, the LSP recombination that we do observe occurs at or near these LSP boundaries, which is consistent with our results for the variable boundaries of multiple *S. paradoxus* introgressions [19]. Finally, two SK1 chromosome I DSB hotspots (181,362 and 184,849) [20], which are located within the region corresponding to LSP2 (Figure 6), and two SK1 chromosome VI DSB hotspots (204,612 and 207,642), which are located adjacent to LSP11 (Figure 7), are not present in EM93, consistent with reduced recombination due to LSP heterozygosity.

If heterozygous non-sub-telomeric LSPs strongly reduce recombination, a hypothesis is that if approximately the same number of recombination events must be generated per chromosome to assure proper segregation then new, compensatory recombination hotspots may be generated. Consistent with this hotspot hypothesis, the midpoint of hotspot 5, which is the hottest CO hotspot in EM93, is centered at chromosome I position 193,116, which is between the heterozygous LSP2 and LSP3 regions. Similarly, the midpoint of hotspot 6, which is the second hottest NCOGC hotspot in EM93, is centered at chromosome I position 204,759 (Figure 6, Table S7), which is between the heterozygous LSP3 and LSP4 regions. Thus, the positions of hotspots 5 and 6 are consistent with heterozygous LSPs generating novel, nearby recombination hotspots.

The numbers of chromosome I (Figure 6) and chromosome VI (Figure 7) recombination hotspots and number of CO events per meiosis in EM93 and YJM789/S288C are similar. As shown in Figure 6, EM93 hotspots 3 and 4 overlap with, or are very near, the previously described *CDC19* and *CYS3* hotspots [21], respectively, which in turn overlap with, or are very near YJM789/S288C hotspots. These shared hotspots are distant from heterozygous LSPs. As also shown in Figure 6, EM93 hotspot 5 overlaps with, or is very near, YJM789/S288C hotspot 7, which is very close to the shared heterozygous LSP3 (EM93)/*S. paradoxus* introgression (YJM789/S288C), consistent with heterozygous LSPs generating novel, nearby recombination hotspots.

On chromosome VI, the classically described *HIS2* (ORF: 203,731–204,738) [22], [23], [24] and *SUP6* (tRNA: 210,607–210,695) [24], [25] recombination hotspots [21] overlap with or are very near the molecularly identified SK1 chromosome VI DSB hotspots 204,612 and 207,642, respectively [20]. EM93 and YJM789/S288C lack the *HIS2* hotspot [10], which in EM93 is adjacent to LSP11, but EM93 hotspot 52 and a nearby YJM789/S288C hotspot overlap with, or are very near, the *SUP6* hotspot (Figure 7). On chromosome VI, EM93 hotspots 48, 49, 50 and 52 overlap with or are very near four YJM789/S288C hotspots [10] (Figure 7).

Despite the similarities listed above, EM93 chromosome I hotspots 1, 2 and 6 do not correspond to any YJM789/S288C chromosome I hotspot and YJM789/S288C hotspots 1, 2, 4 and 6 do not correspond to any EM93 hotspot (Figure 6). Similarly, EM93 chromosome VI hotspots 47 and 51 are not present in YJM789/S288C and one YJM789/S288C chromosome VI hotspot (between 54495 and 56852) is not present in EM93 (Figure 7). Of these hotspots that are unique to EM93 or YJM789/S288C, only EM93 hotspot 6 (centered at 204,623) is near (1,846 bp) a heterozygous LSP. Therefore, while LSP heterozygosity is likely to abolish hotspots, and may consequently generate novel, nearby recombination hotspots, such as EM93 hotspots 5 and 6, other factors are responsible for generating other strain-specific recombination hotspots.

### NCOGC frequency, NCOGCs *vs*. COs and kB/cM

To further examine recombination in EM93, we determined the NCOGC frequencies for all 2,965 polymorphisms. For the 50 polymorphisms with the highest NCOGC frequencies, we then determined the number of COs between the markers flanking these 50 NCOGC polymorphisms, the NCOGC/CO ratios and the kB/cM (Table 1, Table S9). In 120 tetrads, none of the flanking markers underwent NCOGC (Table S9). Both the NCOGC/CO ratios and kB/cM (genome-wide average = 3.52 kB/cM; 50 intervals average = 2.22 kB/cM) varied across a very wide range (Table 1). Because we found heterozygous non-sub-telomeric LSPs affected recombination, we determined the association of distances between the NCOGC polymorphisms and both LSPs and telomeres vs. NCOGC/CO and kB/cM; there were no significant associations (Spearman's rank correlation: LSP-NCOGC SNP distance vs. NCOGC/CO, n = 43, *p-value* = 0.99; LSP-NCOGC SNP distance vs. kB/cM, n = 41, *p-value* = 0.13; telomere-NCOGC SNP distance vs. NCOGC/CO, n = 47, *p-value* = 0.18; telomere-NCOGC SNP distance vs. kB/cM, n = 47, *p-value* = 0.25). Therefore, for these 50 intervals, factors other than LSP or telomere proximity are responsible for the high variation in NCOGC/CO and kB/cM ratios.

**Table 1:** NCOGC frequency, parity vs. non-parity and NCOGCs vs. COs.

Rank	Chr.	Coordinates of NCOGC SNP	SNP-LSP Distance	Total # of NCOGC	1∶3 NCOGC	3∶1 NCOGC	Coordinates of markers flanking NCOGC SNP	Total # of CO	Ratio NCOGC/CO	kb/cM
1	1	207154	4377	24	2	22^1^	202364–224761	4	6.0	2.87
2	16	727489	284786	18	10	8	726624–730555	5	3.6	0.23
3	10	219325	16016	13	13	0^2^	218051–220352	1	13.0	3.06
4	6	35896	19112	12	6	6	32870–36516	2	6.0	3.63
5	9	55057	150587	12	8	4	48869–65509	21	0.6	0.71
6	10	705481	502172	12	6	6	698817–705760	13	0.9	1.23
7	15	987472	85823	12	6	6	985916–989604	8	1.5	0.47
8	8	506124	411190	11	6	5	469077–522577	43	0.3	1.89
9	11	492956	55920	11	7	4	491888–494246	1	11.0	2.56
10	16	426495	10789	11	8	3	396191–428320	23	0.5	3.16
11	6	226159	20245	10	5	5	207830–228481	49	0.2	0.83
12	6	245474	39560	10	4	6	234568–258527	19	0.5	1.38
13	9	166164	39480	10	3	7	164868–167620	1	10.0	3.11
14	15	71614	60585	10	5	5	68376–74107	6	1.7	0.78
15	2	46708	218793	9	3	6	40974–118939	13	0.7	1.06
16	7	167431	234908	9	3	6	165776–171277	1	9.0	3.97
17	8	123594	28660	9	3	6	121713–125757	6	1.5	0.75
18	9	266704	34005	9	5	4	260006–268168	6	1.5	2.68
19	13	168927	NA	9	4	5	168076–174961	3	3.0	0.68
20	15	340372	329343	9	2	7	338394–340732	0	>9.0	>4.76
21	15	830761	242534	9	6	3	801267–831037	38	0.2	1.86
22	3	246792	61433	8	5	3	244165–249279	9	0.9	0.70
23	4	316797	NA	8	3	5	308239–316841	11	0.7	1.87
24	7	293241	109098	8	4	4	286692–293253	1	8.0	15.72
25	8	141666	46732	8	4	4	139384–142275	3	2.7	1.83
26	9	47640	158004	8	4	4	45971–48869	3	2.7	1.34
27	9	96848	108796	8	6	2	91092–99744	8	1.0	1.73
28	11	580437	NA	8	5	3	578505–580474	0	>8.0	>4.64
29	11	630662	NA	8	3	5	628758–631654	8	1.0	0.57
30	13	79932	NA	8	6	2	64448–79936	13	0.6	2.86
31	14	77011	691	8	5	3	72709–77160	3	2.7	3.44
32	14	180729	102603	8	3	5	161254–184130	11	0.7	4.25
33	15	177004	165975	8	2	6	176456–177722	1	8.0	1.32
34	15	602243	471052	8	6	2	601743–603028	5	1.6	0.24
35	16	47122	29610	8	3	5	44104–52999	9	0.9	0.80
36	1	196098	2768	7	7	0^3^	190132–198648	34	0.2	0.42
37	2	193537	71964	7	3	4	191518–198087	9	0.8	0.54
38	2	290464	24277	7	5	2	280672–302383	22	0.3	1.07
39	5	26041	15934	7	5	2	24894–26667	2	3.5	1.38
40	5	43058	32951	7	3	4	39169–46317	8	0.9	1.17
41	7	407444	2787	7	3	4	405424–408707	1	7.0	4.85
42	7	731074	18833	7	4	3	730219–734615	0	>7.0	>2.05
43	8	121713	26779	7	3	4	105511–123594	9	0.8	4.32
44	8	289016	194082	7	3	4	286229–290523	2	3.5	3.34
45	9	268168	32541	7	3	4	266704–272654	3	2.3	1.17
46	9	375196	59180	7	2	5	362705–378374	9	0.8	3.33
47	10	188446	9391	7	4	3	184143–191363	5	1.4	2.07
48	10	316435	113126	7	3	4	313728–319675	9	0.8	0.72
49	11	166676	NA	7	4	3	165239–167774	6	1.2	0.57
50	12	40540	NA	7	3	4	35464–42230	13	0.5	0.94
Total				456	229	230		480	Avg.3.0	Avg.2.12

All SNP (polymorphic marker) coordinates and distances correspond to S288c coordinates and distances. NCOGC = the 50 SNPs exhibiting the most non-crossover associated gene conversion events; CO = crossovers (reciprocal recombination events) between markers flanking each NCOGC SNP; cM = (((3× # non-parental di-type CO tetrads) + (# of tetratype CO tetrads/2))/total # of tetrads) ×100 [57]; cM/kb = cM ÷ distance between markers flanking each NCOGC SNP. NA – Not Applicable: there are no LSPs on the chromosome.

1 *p-value 0.0034*.

2 *p-value 0.0058*.

3 *p-value 0.044*.

Fisher exact test. Parity/non-parity is determined by the segregation and scoring of the S288C∶non-S288C genetic probes.

### NCOGC parity *vs*. non-parity

Although NCOGC parity (i.e. polymorphisms that produce approximately equal numbers of 1∶3 and 3∶1 NCOGCs and are thus unbiased) is most common, exceptional polymorphisms exhibit NCOGC bias or NCOGC non-parity [26]. Because we scored 2,965 polymorphisms in 120 EM93 tetrads, we were in a unique position to assess NCOGC parity vs. non-parity across the genome. Because a high level of NCOGC is necessary to identify deviations from parity, we focused on the 50 polymorphisms that exhibited the highest NCOGC frequencies and then determined the numbers of 1∶3 and 3∶1 (S288C∶non-S288C) NCOGC events at each of these 50 polymorphisms (Table 1).

Of the 50 polymorphisms with the highest NCOGC frequencies, only three polymorphisms exhibited statistically significant NCOGC non-parity (Table 1, NCOGC SNP numbers 1, 3 and 36). Two of these NCOGC non-parity polymorphisms (NCOGC SNP numbers 1 and 36) are on chromosome 1 (Table 1) but are in different hotspots (Table S7) and only one co-conversion was observed; thus most of these NCOGC events were independent. Because of our results showing the effect of heterozygous non-sub-telomeric LSPs on recombination, we determined the locations of these three NCOGC non-parity polymorphisms relative to LSPs and found that all were located within 17 kb of an LSP (Table 1) (*p-value* = *0.0061*, Fisher exact test).

Given this highly significant LSP-NCOGC non-parity polymorphism association, we considered the hypothesis that biased intra-LSP recombination, which might arise from biased intra-LSP DSB formation, was responsible for the observed cases of non-parity NCOGC in linked SNPs. To test this hypothesis, we determined the numbers of CO and NCOGC within LSP2, LSP3 and LSP4 that flank non-parity NCOGC SNP numbers 1 and 36. (As described above, LSP22, which is adjacent to non-parity NCOGC SNP number 3, is one of three Ty4 presence/absence polymorphisms. The three independently segregating copies of Ty4 prohibited examination of LSP22 recombination events by arrays.) No recombination events (CO or NCOGC) were observed within LSP2 (20 markers), LSP3 (3 markers) and LSP4 (3 markers). Therefore, in addition to being low, recombination within LSP2, 3 and 4 was unbiased; that is, intra-LSP recombination bias was not responsible for the non-parity NCOGC of SNP numbers 1 and 36. Instead, we hypothesize that LSP2, 3 and/or 4, and possibly LSP22, have other *cis*-acting effects on DSB formation and/or on repair that result in non-parity NCOGC in linked SNPs.

## Discussion

### Genomic differences between EM93 and S288C

By hybridizing DNA from the segregants of two EM93 tetrads to GENECHIP *S. cerevisiae* Tiling 1.0R Arrays, we generated a broad genomic overview of this natural isolate that is estimated to contribute 88% of the S288C genome [12]. Large regions on one arm of multiple EM93 chromosomes, especially IV(R) and XII(R), were entirely homozygous for S288C-like sequences (Figure 3). The locations of these homozygous regions suggest that homozygosity may have resulted from mitotic recombination.

Sequence analysis of the *S. cerevisiae* clinically derived strain YJM789 provided evidence for an introgression of a chromosome I region from the closely related yeast, *S. paradoxus* [14]. *S. paradoxus* and *S. cerevisiae* share similar environments and hybrids have been found in nature [27], [28]. We provided further support for *S. cerevisiae* and *S. paradoxus* hybrids being formed and that the progeny of such hybrids are likely to be responsible for the introduction of *S. paradoxus* DNA into the *S. cerevisiae* genome [19]. Our finding that EM93 is heterozygous for an introgression similar to that present in YJM789 suggests a relatively recent common ancestor for the fig isolate EM93 [12] and the clinically derived strain YJM789.

LSP14 on chromosome VII is homozygous; thus, the corresponding region in S288C does not originate from EM93. This is consistent with S288C not being a direct offspring of EM93, but instead being derived via a series of crosses with additional strains [12]. The 5′ and 3′ sequences LSP14 in EM93 are almost identical (99%) to that present in the clinically derived strain YJM789.

Based on the hybridization profiles, many non-sub-telomeric LSPs correspond to the locations of Ty-elements or free LTRs in S288C. Ty-elements, which compose about 3% of the S288C genome, are flanked by long-terminal-repeats (LTR) that can induce LTR-LTR recombination leading to gain or loss of Ty-elements. We confirmed that five heterozygous, Ty-associated non-sub-telomeric LSPs in EM93 are due to the presence/absence of Ty3 and Ty4.

### Distribution and frequency of recombination events

Meiosis is a specialized form of cell division that involves one round of chromosome replication followed by two rounds of segregation, producing four haploid spores with new combinations of alleles [29]. In most organisms, like *S. cerevisiae*, DNA DSBs are introduced into the genome by the topoisomerase-like Spo11 protein following pre-meiotic S-phase [30], [31]. These DSBs are repaired almost solely by homologous chromosome repair pathways that result in either CO or NCOGC recombination products [32], [33]. Genetic recombination, which occurs at high levels during meiosis, plays an important role in boosting genetic diversity in the next generation and ensuring proper chromosome segregation [29], [34], [35], [36]. Chromosomes lacking DSBs have significantly higher rates of missegregation, which could lead to aneuploid gametes [15], likely causing spore death in *S. cerevisiae*. Therefore, it is not surprising that most eukaryotes have mechanisms to control DSBs, leading to at least one recombination event per chromosome, often referred to as the obligate chiasma. Consistent with the obligate chiasma hypothesis [15], and despite the genotyping array covering only 77% of the EM93 genome, we found that 99.7% of the chromosomes investigated showed evidence of recombination events.

When a CO occurs in one region, the likelihood that a CO will occur in an adjacent region is reduced; this is referred to as CO interference [37]. The power of interference decreases as a function of distance along the chromosome [16], [38]. Consistent with this, we observed clear evidence of CO interference (Table S5 and Table S6). The frequency and location of recombination events is also influenced by chromatin structure, which affects DSB formation. Consequently, recombination is reduced in regions with compacted chromatin and increased in regions with open chromatin [39], [40], [41], [42], [43], [44]. Similar to other studies [5], [18], EM93 showed an approximately 70% reduction of recombination events within the centromere region (∼20 kB), consistent with centromeres being recombination cold spots.

In addition to centromeres, we determined that heterozygous non-sub-telomeric LSPs reduce recombination in adjacent regions by approximately 75%. This suggests that heterozygous non-sub-telomeric LSPs not only repress recombination but also change the recombination pattern. While the mechanism is unclear, this heterozygous sub-telomere proximal LSP repression of recombination is similar to previous cases where heterozygous sequences alter recombination [45].

In contrast to heterozygous non-sub-telomeric LSPs, we show that heterozygous sub-telomeric LSPs do not repress recombination. By allowing recombination between homologous sub-telomeres, proper chromosome segregation may be promoted. Alternatively, allowing recombination between non-homologous sub-telomeres will promote gain-loss of sub-telomeric genes that may be beneficial. Indeed, this is consistent with *S. cerevisiae* chromosomes being organized into two structural domains, with the central core containing essential genes and the sub-telomeric regions containing non-essential genes that are subject to rearrangements [46], [47].

### Recombination hotspots

Recombination (or, more accurately, DSB formation) studies in strains such as SK1 [20], which lack heterozygosities, differ both technically and genetically with recombination studies in multiply heterozygous strains, such as YJM789/S288C [10] and isolates such as EM93 (this study). For example, multiple heterozygosities allow one to distinguish between NCOGC and CO events. Thus, similar to YJM789/S288C [10], we find that in EM93 NCOGC + CO recombination hotspots are outnumbered by CO hotspots that in turn are outnumbered by NCOGC hotspots (Table S7). In addition, for 50 intervals, we find very high variation in NCOGC/CO and kB/cM ratios (Table 1). Despite the technical and genetic differences between DSB studies in homozygous strains and recombination studies in multiply heterozygous strains, the 203 EM93 hotspots that we identified (Figure S7 and Table S7) include many previously described recombination hotspots, such as *ARG4-DED81*, *CYS3*, *ARE1/IMG1*, *CDC19*, *THR4*, *LEU2-CEN3* [21].

In addition to previously described and, hence, conserved recombination hotspots, we also found many novel, strain-specific recombination hotspots, even between YJM789/S288C and EM93 that are related and where similar techniques were used to measure recombination. Specifically, in EM93 where we examined 77% of the genome, we identified 117 CO hotspots (Table S7). In the corresponding regions of YJM789/S288C, Mancera et al. [10] identified 80 CO hotspots. While 32 CO hotspots were present in both EM93 and YJM789/S288C, 48 were unique to YJM789/S288C and 85 were unique to EM93.

Our results are consistent with non-sub-telomeric LSP heterozygosity causing the loss of specific recombination hotspots in EM93. Our results also suggest that non-sub-telomeric LSP heterozygosity may promote the formation of novel, nearby recombination hotspots. For example, we found that the hottest CO hotspot was located between chromosome I LSP2 and LSP3, close to the boundary of LSP2. Therefore, whether in natural isolates where heterozygous LSPs appear to be common [19], such as EM93, or in crosses between unrelated strains, such as YJM789/S288C [10], heterozygous LSPs are likely to be an important feature affecting recombination.

However, the absence of heterozygous LSPs near most of the strain-specific recombination hotspots, as well as the lack of association between LSP distances *vs*. NCOGC frequencies as well as NCOGC/CO and kB/cM ratios (Table 1), suggests that additional factors affect recombination and the locations of recombination hotspots. Chromatin structure and promoter activity are known to affect recombination [42], [43], [44]. Therefore, some strain-specific recombination hotspots may be epigenetic while other strain-specific recombination hotspots may be due to polymorphic transcription factors, which presumably act in *trans*, and/or to polymorphic promoters, which presumably act in *cis*. Determining the mechanistic bases of strain-specific hotspots, both LSP- and non-LSP-dependent, will be highly informative as to the mechanistic bases of all recombination hotspots.

### NCOGC parity vs. non-parity

On average, the frequency of gene conversion events is approximately 4% [48]. For most polymorphisms, gene conversion parity is observed; that is, the number of 1∶3 gene conversion events is approximately equal to the number of 3∶1 gene conversion events [26]. Consistent with these classical data, 47 of the 50 EM93 high NCOGC polymorphisms exhibited parity. However, specific polymorphisms exhibit significant deviations from parity [49], [50], [51]. Of 50 EM93 polymorphisms with the highest levels of NCOGC, three showed statistically significant non-parity, all of which were located less than 17 kB from a heterozygous LSP. Thus, our results identify a novel effect of some LSPs on recombination – non-parity. Having excluded the biased intra-LSP recombination hypothesis, one hypothesis is that some heterozygous LSPs bias heteroduplex repair or induce/repress DSB formation on one homolog, possibly by *cis* effects on chromatin structure or promoter activity outside of the LSP, which results in NCOGC non-parity.

## Materials and Methods

### Strains, Media, DNA extraction, and Polymerase chain reaction

EM93 [12] was obtained from E. Winzeler. Standard yeast media was prepared as described previously [52], [53]. Sporulation, tetrad dissection, and germination of EM93 ascospores were done using standard methods [54]. Genomic DNA was extracted from 50 mL overnight YEPD cultures using QIAGEN Genomic tip 100/G Kits (Cat. no. 10243) according to the manufacturer's instructions. Polymerase chain reactions (PCR) were done using the High-Fidelity DNA Polymerase, iProof (BioRad, #172–5301), according to the manufacturer's protocol. Sequencing was performed by the Duke University Medical Center sequencing facility (http://cancer.duke.edu/dna) using the Perkin Elmer Dye Terminator Cycle Sequencing system with AmpliTaq DNA Polymerase combined with ABI 3730, 3100 PRISM DNA, and BigDyeTMv1.1 terminator sequencing chemistry. Oligonucleotides used in this study are listed in Table S1.

### Affymetrix Tiling Array hybridization and genotyping

Heterozygous and homozygous polymorphisms in the EM93 genome were identified by genotyping each of the segregants of two EM93 tetrads using GeneChip® *S. cerevisiae* Tiling 1.0R Arrays (Affymetrix). Ten micrograms of DNA were digested with 1 U of DNase I (New England Biolabs) in 1× DNase I reaction buffer at 37°C for 2 min to obtain fragments of about 50 bp. DNase I was heat inactivated at 95°C for 20 min and the fragmented DNA was end-labeled by incubation at 37°C for 1 h with 20 U of terminal deoxynucleotidyl transferase (New England Biolabs) and 1 nmol of biotin-11-ddATP (Perkin Elmer) in 1× NEBuffer 4 (New England Biolabs). After inactivation of the terminal deoxynucleotidyl transferase by incubation at 75°C for 25 min, the target DNA was hybridized onto GeneChip® *S. cerevisiae* Tiling 1.0R Arrays (Affymetrix), as described previously [13]. The arrays were scanned using an Affymetrix scanner at 0.7 µm resolution and an average intensity at each oligonucleotide feature was calculated based on the hybridization intensity of the 9 central pixels using the GeneChip® Operating Software (Affymetrix). The MIAME compliant Affymetrix Tiling Array data has been deposited in the ArrayExpress database (accession number: E-MEXP-3246).

### Affymetrix Tiling Array data analysis

The hybridization intensity data were background corrected with the RMA algorithm, quantile normalized, and log_2_ transformed with the aroma affymetrix package [55] in R v2.9.0 [56]. Large sequence polymorphisms (LSPs; deletions or highly polymorphic regions ≥500 bp) were identified based on low levels of the hybridization intensities of overlapping oligonucleotide features using the Integrated Genome Browser software (Affymetrix). A complete list of identified LSPs is shown in Table S2.

Based on hybridization intensities, there were over 45,000 segregating Tiling Array probes in EM93 (Table S3). The hybridization intensities for each probe for the eight segregants originating from two tetrads were grouped and ranked and from the ∼45,000 segregating probes, 6,000 were selected based on signal intensity, fold differences (selected probes had an average difference of 9.9), level of significance (*student's t-test*, *P<0.05*), and genome distribution (based on the probes' positions according to S288C genomic coordinates).

### Combimatrix array

The selected probes were transferred to a Combimatrix 1×12K custom array for further validation. Genomic DNA from the eight segregants, previously analyzed on the Tiling Array, was hybridized to the Combimatrix array. DNA preparation, labeling and data analysis were done as previously described [19]. Digested and labeled DNA was hybridized to a custom made 1×12K Combimatrix microarray following the standard protocols from Combimatrix for hybridization, washing, and staining. From the 6,000 re-tested probes, we selected a final set of 2,965 probes, using the same criteria described for the Tiling Array probes above (Table S3), which were then transferred to a custom made 8×15K Agilent microarray carrying five copies of each of the 2,965 probes.

### Agilent genotyping array

Four hundred and eighty segregants from 120 EM93 tetrads were hybridized to the custom Agilent microarrays (the custom genotyping array is deposited in the ArrayExpress database, accession number: E-TABM-1174). The final genotyping protocol was as follows: 18 micrograms of genomic DNA from each segregant was digested using 1.5 U DNase I (NEB, M0303S) for 1.40 min at 37°C in 1× DNaseI reaction buffer generating approximately 50 bp long fragments. Nine micrograms of fragmented DNA was 3′-labeled using 20 U of terminal deoxynucleotidyl transferase (NEB M0315S) and 1.5 nmol Cy5-dATP (PerkinElmer NEL593001ER) in 1×NEBuffer (New England Biolabs). Seven micrograms of end-labeled DNA was prepared for hybridization according to the manufacturer's protocol (Array-based CGH for Genomic DNA Analysis, Step3: Preparation of Labeled Genomic DNA for Hybridization). Prepared DNA was hybridized for 18 h at 45°C, washed using Agilent CGH-washing buffer 1 for 5 min at room temperature and subsequently washed in Agilent-CGH washing buffer 2 for 3 min at 45°C. Arrays were scanned using an Axon GenePix 4000B scanner at 5 µm resolution; data were extracted using GenePix Pro V6.0. The MIAME compliant Agilent genotyping array data has been deposited in the ArrayExpress database (accession number: A-MEXP-2076). Sequences of the Agilent array genotyping probes are given in Table S10 (probes).

### Genotyping and Recombination events

We genotyped 480 segregants from 120 tetrads using 2,965 genetic markers on a custom Agilent genotyping array. For each segregant, DNA was isolated, prepared, labeled and hybridized as described above. The hybridization intensity was background corrected using the mean-normalization method, examples of which are shown in Figure S1. Using the mean hybridization intensity from the five probe replicates for all the segregants for each marker and K-mean cluster analysis (XLMiner, Cytel software Corporation, expecting two clusters), a genotype call for each genetic marker in each segregant was made (S288C-like or non-S288C). After grouping genotype calls by tetrad, adjacent markers that had both segregated 2∶2 and had undergone a reciprocal recombination event were designated as crossovers (COs). After grouping genotype calls by tetrad, individual markers that segregated 1∶3 or 3∶1 were designated as gene conversions (GCs). Each GC was then designated as either a crossover associated GC (COAGC), if the markers flanking that GC had undergone a CO, or as a non-crossover gene conversion (NCOGC), if the markers flanking that GC had not undergone a CO. To determine recombination hot-spots and cold-spots, the observed number of events per interval (i.e. between two genetic markers) was divided by the expected number of events. The expected number of events, which assumes a random genome-wide distribution of 87.1 CO/meiosis and 30.8 NCOGC/meiosis, was obtained by calculating the number of events per Mega-base (6.5 CO/Mb/meiosis and 2.6 NCOGC/Mb/meiosis). For closely spaced markers, the interval size was adjusted to at least 2 kb (i.e. such intervals contained ≥2 markers) to avoid single recombination events being falsely scored as recombination hotspots. When two or more contiguous intervals (each ≥2 kb) were scored as above the hotspot threshold, these intervals were counted as one hot spot; the new hotspot midpoint was then calculated based on the midpoint of the contiguous intervals. The 480 segregant genotypes are given in Table S10 (genotypes).

## Supporting Information

Figure S1 **Examples of genotyping data.** The fluorescent intensity for each array was normalized using the mean-normalization method. For each marker, the mean value of the five replicates was calculated. Above graphs show the hybridization intensities for eighteen full tetrads (n = 72) for twelve randomly selected markers. The hybridization intensities are plotted in LOG_10_ scale.
(TIFF)

Figure S2 **Sequence comparison of a transgressed** ***Saccharomyces paradoxus*** **DNA fragment.** EM93 is heterozygous for a *S. paradoxus* sequence on chromosome I similar to that present in YJM789. Above is a comparison between the sequences obtained from segregants 2A and 2C to S288C and YJM789. Alignments were generated by using NCBI's Blasting function.
(TIFF)

Figure S3 **Sub-telomeric LSPs.** Graphs show the hybridization intensities for four segregants originating from a full EM93 tetrad. Graphs show sub-telomeric LSPs present on: (A) Chr I (LSP 1), (B) Chr I (LSP 4), (C) Chr III (LSP 7), (D) Chr V (LSP 8), (E) Chr VI (LSP 9), (F) Chr VII (LSP 14), (G) Chr IX (LSP 20), (H) Chr X (LSP 21), (I) Chr XIV (LSP 23), (J) Chr XV (LSP 24), (K) Chr XV (LSP 25), and (L) Chr XVI (LSP 26) in EM93. For LSP number, see Table S1. Graphs are created using a moving average of the hybridization intensities of the probes that have a unique hit in the S288C genome with a window size of 200 probes.
(TIFF)

Figure S4 **Polymorphisms associated with Ty3.** (A) EM93 lacks Ty3 on chromosome VII. Sequence analysis across the Ty3-element showed that EM93 is missing the sequence between nucleotide 707,199 and 712,548. (B) EM93 is heterozygous for Ty3 on chromosome IX and sequencing analysis showed that the region missing was between nucleotides 205,556 and 210,596. Also see figure 2 and Table S1 LSP 13 and 17 for further information. Alignments shown above were generated by using NCBI align function. Underlined sequences indicate the predicted junction.
(TIFF)

Figure S5 **Ty4 genotyping.** The hybridization profile obtained from the Tiling Array analysis showed that segregant 2D lacked hybridization to probes specific for Ty4. Also see Figure 2C–D and Table S1 LSP 15, 22, and 27 for further information. By using both internal and Ty-specific primers pairs (indicated with arrows) we showed that EM93 is heterozygous for all three Ty4-elements, supporting the hybridization profiles obtained from the Tiling Array.
(TIFF)

Figure S6 **LSP hybridization.** Graphs show the hybridization intensities for four segregants originating from an EM93 tetrad for a subset of identified LSPs: (A) LSP 3, (B) LSP 5, (C) LSP 6, (D) LSP 10, (E) LSP 11, (F) LSP 12, (G) LSP 16, (H) LSP 18 and (I) LSP 19. See Table S2 for further information. Graphs are created using a moving average of the hybridization intensities of the probes that have a unique hit in the S288C genome with a window size of 200 probes.
(TIFF)

Figure S7 **Frequency and distribution of recombination events in EM93.** The distribution and frequency of CO and NCOCO events across the heterozygous regions of the EM93 genome was determined by counting the respective events between each adjacent marker. The counts were then adjusted for the size of the interval. By dividing the number of CO and NCOGC for each interval with the expected frequency of the respected event (CO∶6.5/Mb/meiosis, NCOGC: 2.6/Mb/meiosis) produced a recombination score. Dashed line indicates the expected frequency if considering a homogeneous distribution (frequency = 1). Centromere is indicated with a (▾).
(TIFF)

Figure S8 **Hotspot distance in EM93.** The distance between hotspots (CO and NCOGC) across the EM93 genome was determined by using the midpoint for each interval associated with a hotspot. The average distance is 45.2 kb.
(TIFF)

Table S1 Oligonucleotides used in this study.
(DOC)

Table S2 **List of LSPs in EM93.** Large sequence polymorphisms (LSPs; ≥500 bp) were identified based on low levels of the hybridization intensities of overlapping oligonucleotide features using the Integrated Genome Browser software (Affymetrix). A complete list of positions of identified LSPs is shown above. The association of LSPs with Ty3, Ty4, other Ty/LTR elements (potentially (Ty1), (Ty2) or (LTR) present in S288C), sub-telomeric regions (ST), *S. paradoxus* homology (*S.p*), or non-sub-telomeric (non-ST; all Ty and LTR associated LSPs are also non-ST) is also indicated above (Assoc. with). * (*KIN3*, *CDC15*, *YAR019W-A*, *ARS110*, *PAU7*, *YAR023C*, *SUP56*, *tS(AGA)A*, *YARWsigma1*, *YARWdelta6*, *UIP3*, *YAR028W*, *YAR029W*, *PRM9*, and *MST29*).
(DOC)

Table S3 Tiling Array probes that segregate 2∶2 in EM93.
(DOC)

Table S4 Summary of EM93 genotyping markers.
(DOC)

Table S5 Crossover interference on chromosome XI.
(DOC)

Table S6 Crossover interference on chromosome XV.
(DOC)

Table S7 Location of Hotspots.
(DOC)

Table S8 Recombination in intervals adjacent to LSPs.
(DOC)

Table S9 Non-Crossover Gene Conversion frequency and tetrad segregation.
(DOC)

Table S10 **Segregant Genotypes and Genotyping Probes (Excel file). Genotyping probes (n = 2965):** Nr = Probe number. Chr = Chromosome number; Position = Nucleotide number (S288C numbering) of the first probe nucleotide; Sequence = Probe sequence. **Segregant Genotypes:** For EM93 tetrads 1–125 (segregants 1–480), genotype calls: 1 = S288C-like genotype and 10 = non-S288C-like genotype; marker location = chromosome #∶Nucleotide number (S288C numbering) of the first genotyping probe nucleotide.
(XLSX)
