# Supplementary figures and images for: Genomic Structure of and Genome-Wide Recombination in the Saccharomyces cerevisiae S288C Progenitor Isolate EM93
Source: PLoS One. 2011 Sep 26;6(9):e25211. doi: 10.1371/journal.pone.0025211 (PMC3180460; doi:10.1371/journal.pone.0025211)

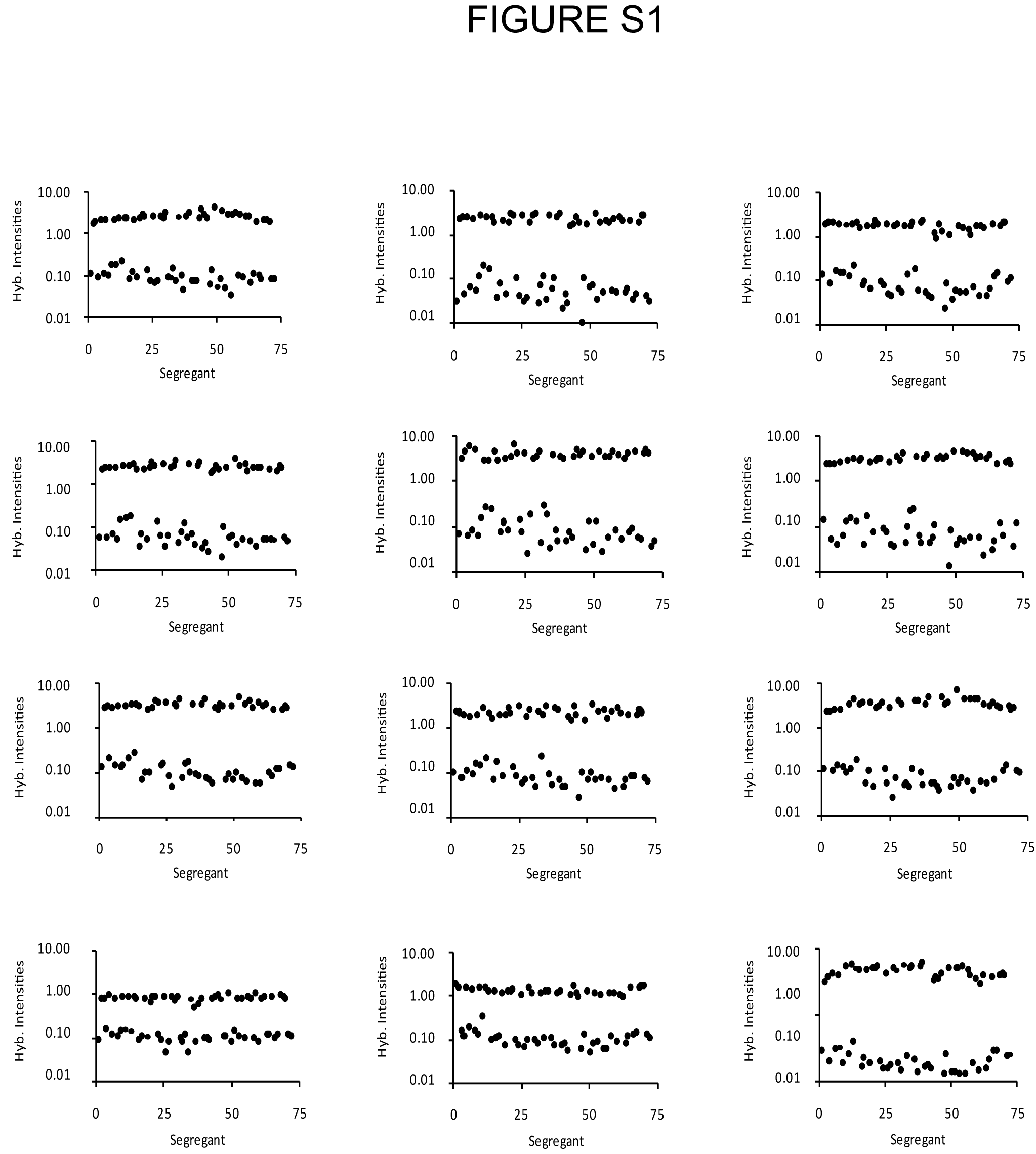

Supplement: Figure S1 — Examples of genotyping data. The fluorescent intensity for each array was normalized using the mean-normalization method. For each marker, the mean value of the five replicates was calculated. Above graphs show the hybridization intensities for eighteen full tetrads (n = 72) for twelve randomly selected markers. The hybridization intensities are plotted in LOG10 scale. (TIFF) [file pone.0025211.s001.tiff]

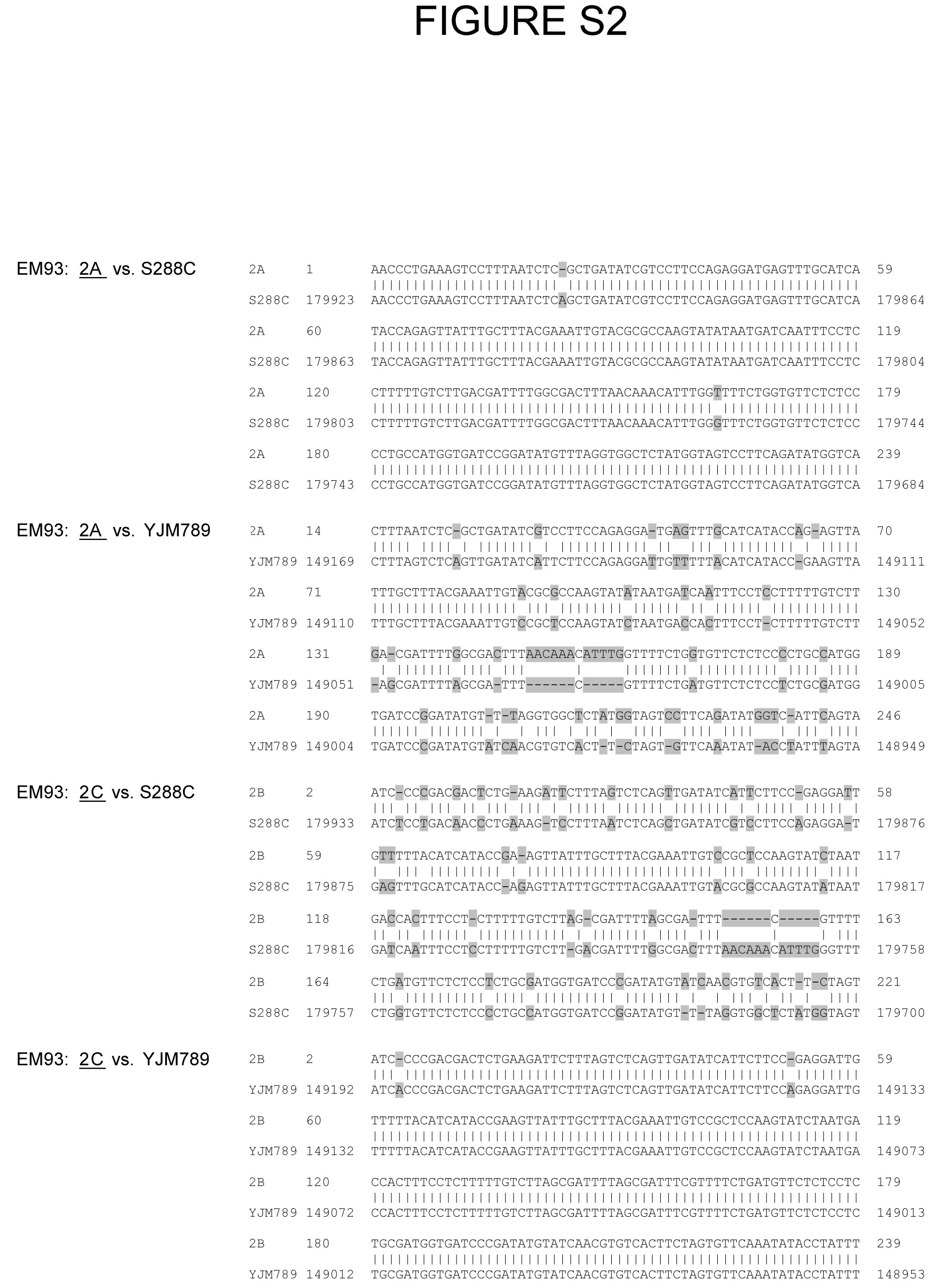

Supplement: Figure S2 — Sequence comparison of a transgressed Saccharomyces paradoxus DNA fragment. EM93 is heterozygous for a S. paradoxus sequence on chromosome I similar to that present in YJM789. Above is a comparison between the sequences obtained from segregants 2A and 2C to S288C and YJM789. Alignments were generated by using NCBI's Blasting function. (TIFF) [file pone.0025211.s002.tiff]

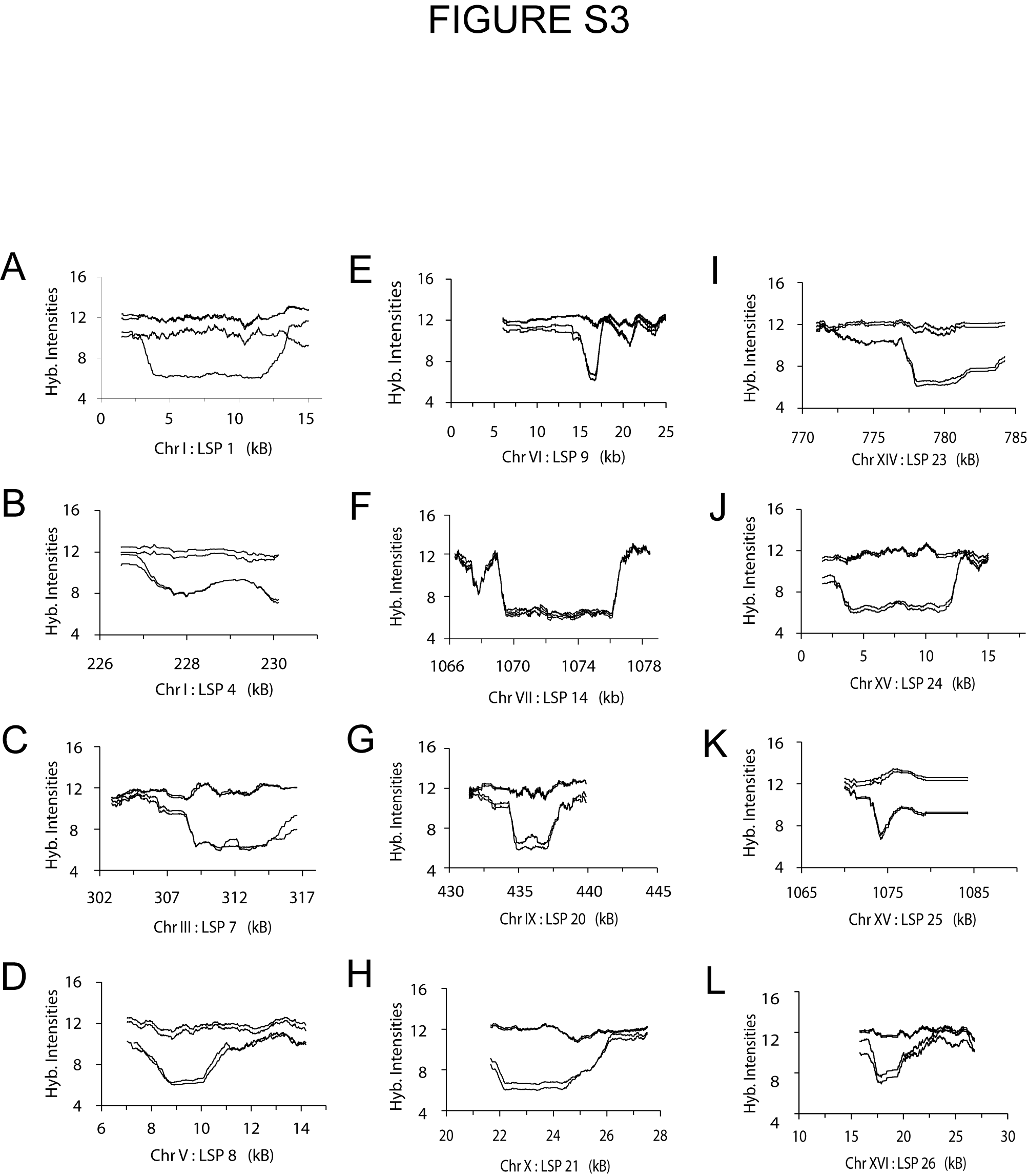

Supplement: Figure S3 — Sub-telomeric LSPs. Graphs show the hybridization intensities for four segregants originating from a full EM93 tetrad. Graphs show sub-telomeric LSPs present on: (A) Chr I (LSP 1), (B) Chr I (LSP 4), (C) Chr III (LSP 7), (D) Chr V (LSP 8), (E) Chr VI (LSP 9), (F) Chr VII (LSP 14), (G) Chr IX (LSP 20), (H) Chr X (LSP 21), (I) Chr XIV (LSP 23), (J) Chr XV (LSP 24), (K) Chr XV (LSP 25), and (L) Chr XVI (LSP 26) in EM93. For LSP number, see Table S1. Graphs are created using a moving average of the hybridization intensities of the probes that have a unique hit in the S288C genome with a window size of 200 probes. (TIFF) [file pone.0025211.s003.tiff]

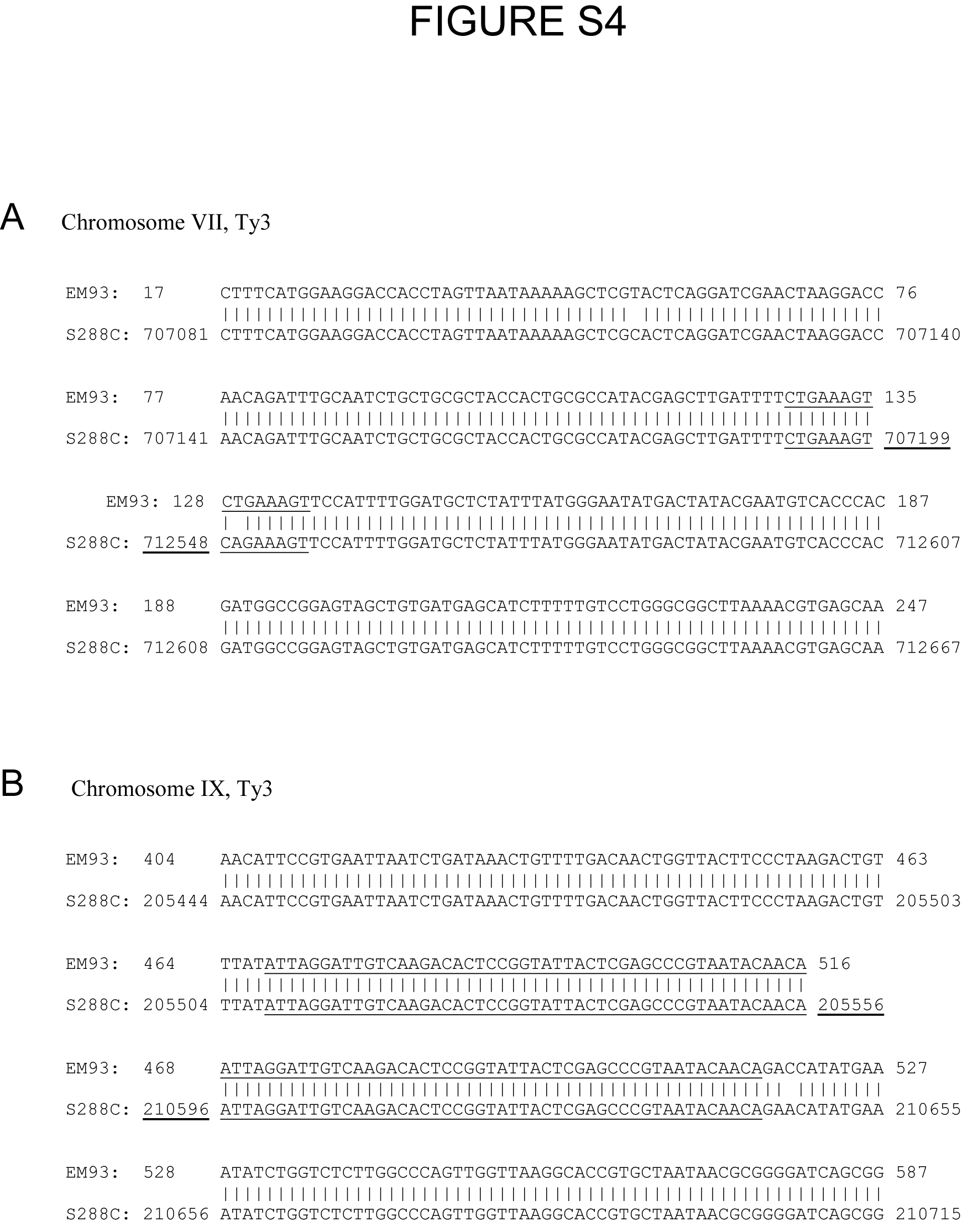

Supplement: Figure S4 — Polymorphisms associated with Ty3. (A) EM93 lacks Ty3 on chromosome VII. Sequence analysis across the Ty3-element showed that EM93 is missing the sequence between nucleotide 707,199 and 712,548. (B) EM93 is heterozygous for Ty3 on chromosome IX and sequencing analysis showed that the region missing was between nucleotides 205,556 and 210,596. Also see figure 2 and Table S1 LSP 13 and 17 for further information. Alignments shown above were generated by using NCBI align function. Underlined sequences indicate the predicted junction. (TIFF) [file pone.0025211.s004.tiff]

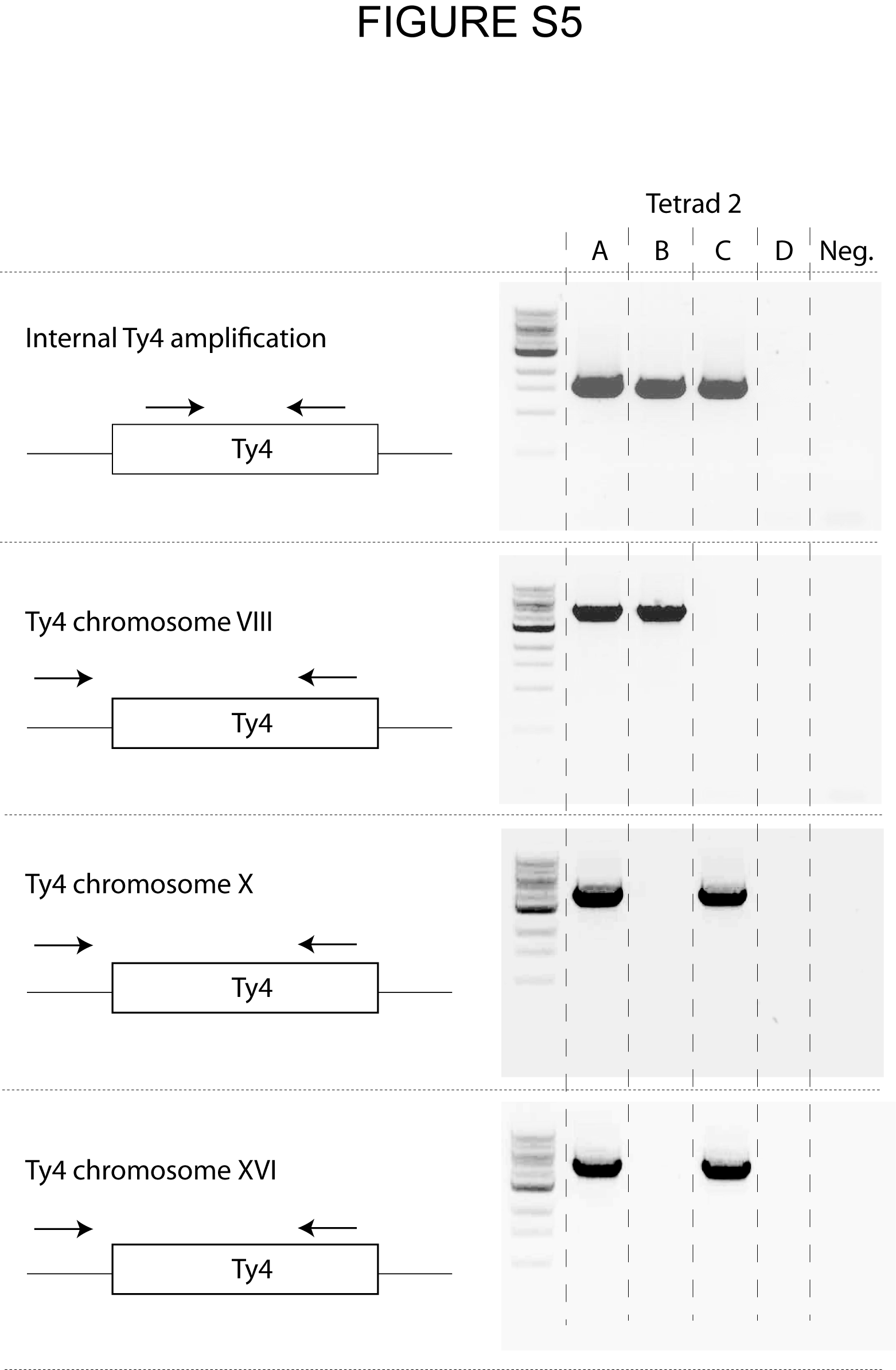

Supplement: Figure S5 — Ty4 genotyping. The hybridization profile obtained from the Tiling Array analysis showed that segregant 2D lacked hybridization to probes specific for Ty4. Also see Figure 2C–D and Table S1 LSP 15, 22, and 27 for further information. By using both internal and Ty-specific primers pairs (indicated with arrows) we showed that EM93 is heterozygous for all three Ty4-elements, supporting the hybridization profiles obtained from the Tiling Array. (TIFF) [file pone.0025211.s005.tiff]

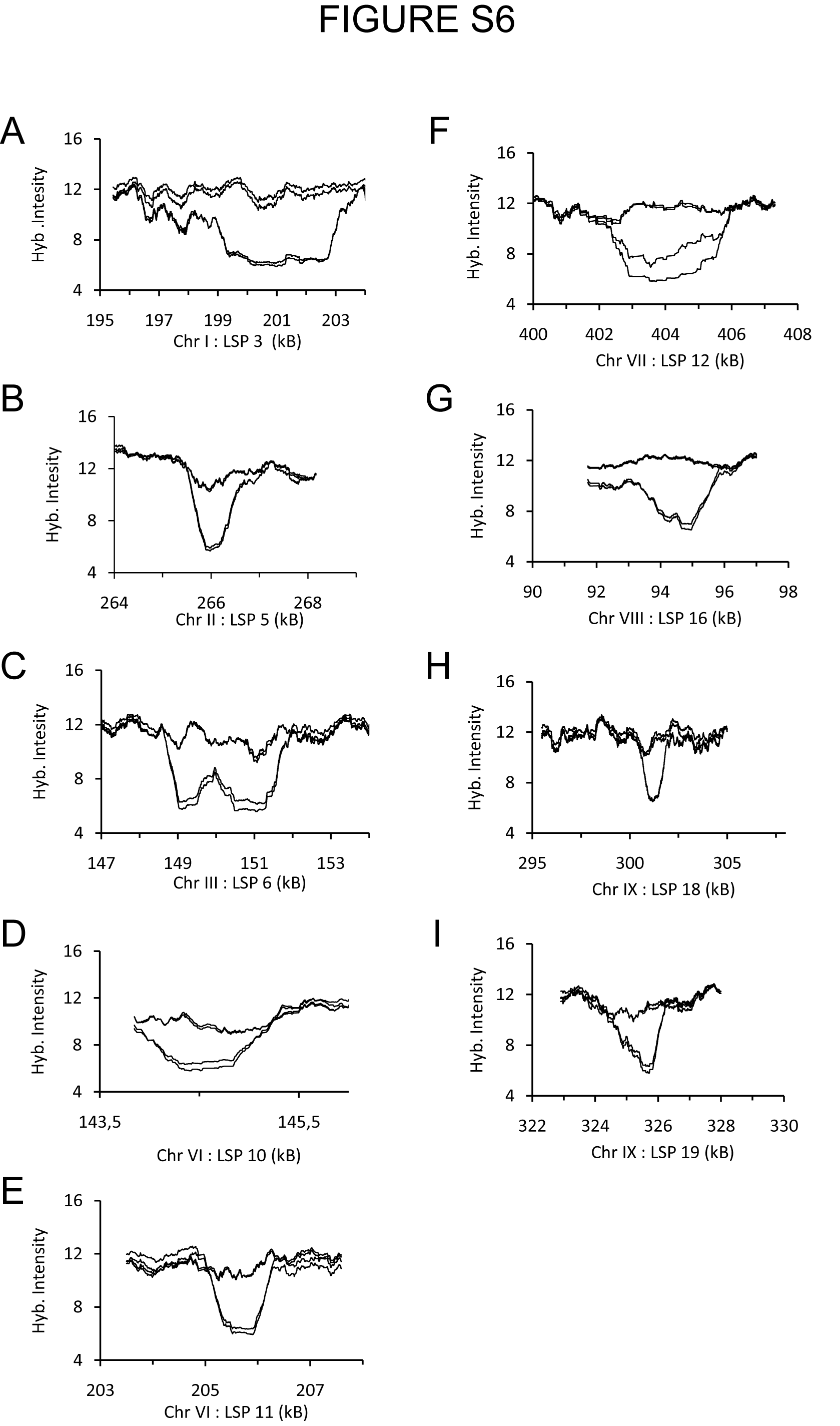

Supplement: Figure S6 — LSP hybridization. Graphs show the hybridization intensities for four segregants originating from an EM93 tetrad for a subset of identified LSPs: (A) LSP 3, (B) LSP 5, (C) LSP 6, (D) LSP 10, (E) LSP 11, (F) LSP 12, (G) LSP 16, (H) LSP 18 and (I) LSP 19. See Table S2 for further information. Graphs are created using a moving average of the hybridization intensities of the probes that have a unique hit in the S288C genome with a window size of 200 probes. (TIFF) [file pone.0025211.s006.tiff]

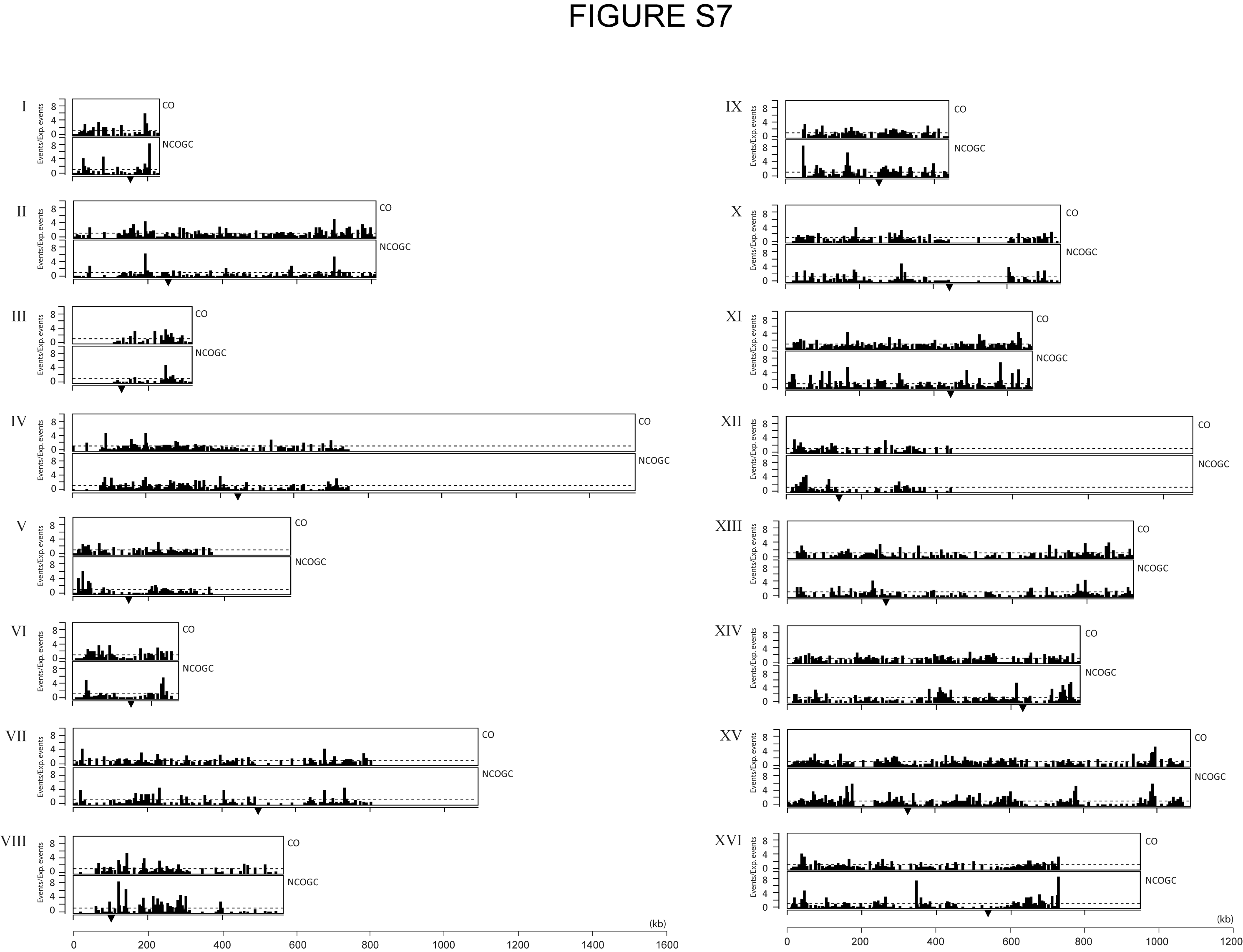

Supplement: Figure S7 — Frequency and distribution of recombination events in EM93. The distribution and frequency of CO and NCOCO events across the heterozygous regions of the EM93 genome was determined by counting the respective events between each adjacent marker. The counts were then adjusted for the size of the interval. By dividing the number of CO and NCOGC for each interval with the expected frequency of the respected event (CO∶6.5/Mb/meiosis, NCOGC: 2.6/Mb/meiosis) produced a recombination score. Dashed line indicates the expected frequency if considering a homogeneous distribution (frequency = 1). Centromere is indicated with a (▾). (TIFF) [file pone.0025211.s007.tiff]

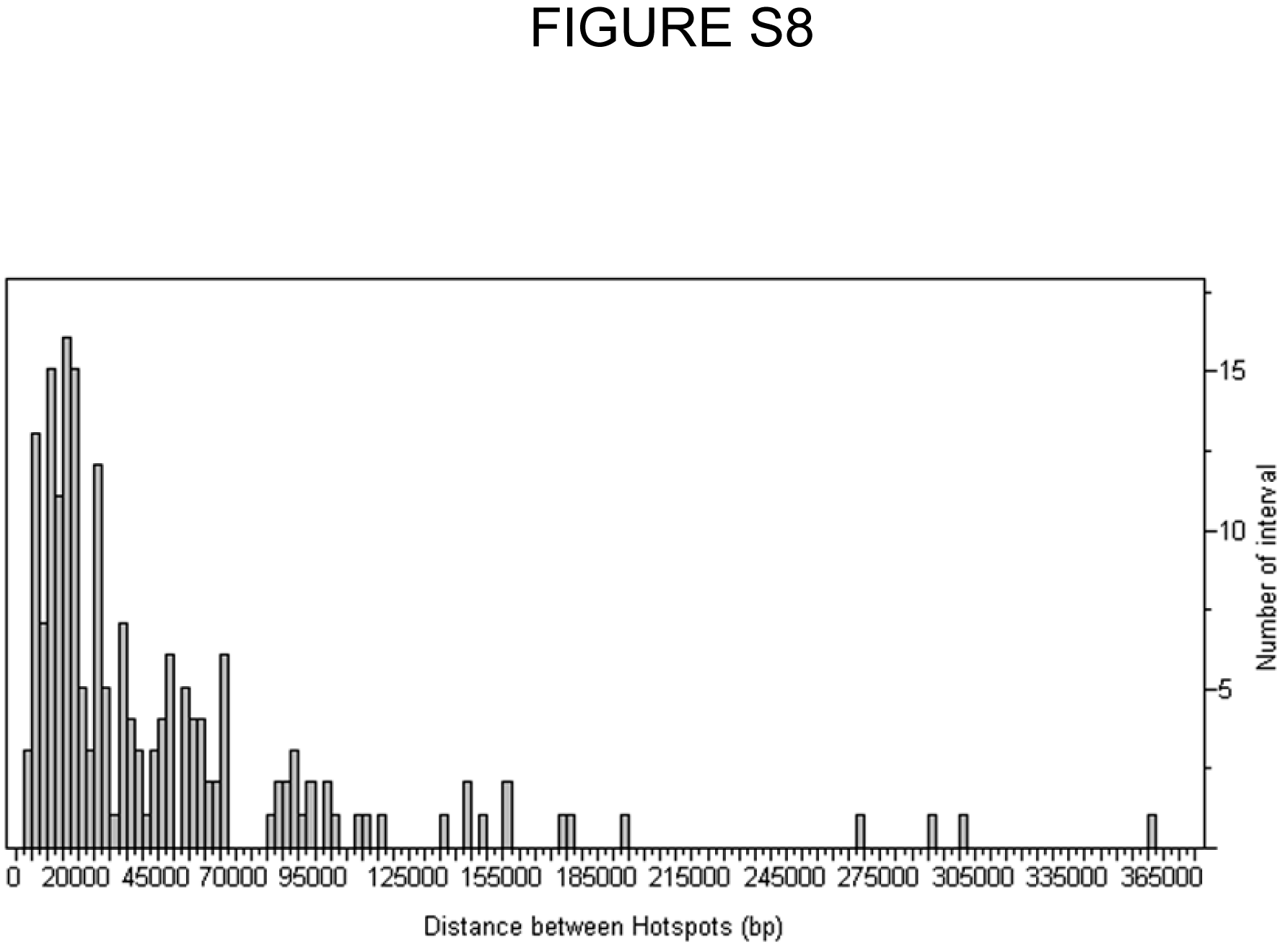

Supplement: Figure S8 — Hotspot distance in EM93. The distance between hotspots (CO and NCOGC) across the EM93 genome was determined by using the midpoint for each interval associated with a hotspot. The average distance is 45.2 kb. (TIFF) [file pone.0025211.s008.tiff]
