# Supplementary material for: Genomic Structure of and Genome-Wide Recombination in the Saccharomyces cerevisiae S288C Progenitor Isolate EM93
Source: PLoS One. 2011 Sep 26;6(9):e25211. doi: 10.1371/journal.pone.0025211 (PMC3180460; doi:10.1371/journal.pone.0025211)
Supplement: Table S1 — Oligonucleotides used in this study. (DOC) [file pone.0025211.s009.doc]

**TABLE S1**

Oligonucleotides used in this study

| **Name** | **Sequence** |
| --- | --- |
| Chr. I *S. Paradoxus* For. | AAATCAAGTTTATTCTCTTCACAACAA |
| Chr. I *S. Paradoxus* S288C seq. Rew | GATCTCCTGACAACCCTGAAAGTC |
| Chr. I *S. Paradoxus* YJM789 Seq. Rew | AATCACCCGACGACTCTGAAGATT |
| TY3 Chr.7 For. | TAAACGCAGGTTGCGAATTG |
| TY3 Chr.7 Rew. | AAGAGCCCTTTCAGAAACAAA |
| TY3 Chr.9 For. | AGCGTTGTGTGCGTAATTGT |
| TY3 Chr.9 Rew. | TCACCATTTAGCGCACAGAA |
| TY4-LTR For. | TTAGGCCTGACTCGACCAATT |
| TY4-LTR Rew. | ATCCAAGTCCCCTTCTAGGTT |
| TY4 Chr. 8 For. | TGTTGCCTGCCCTTTCTTAT |
| TY4 Chr.8 Rew. | CAGTGGCCATTAAGCAAATGA |
| TY4 Chr.10 For. | TGTTTTCTGCTGACCCTCCCT |
| TY4 Chr.10 Rew. | AAAGCGACCGTGTTCAGTCCT |
| TY4 Chr.16 For. | TGGGATTGAATTTTGGGAGAA |
| TY4 Chr.16 Rew. | TCGCACTCAGGATCGAACTAA |
| LSP # 14 For. | TTCGCTGGAACAGTGATTGATGACC |
| LSP # 14 Rew. | CTTAAGAGATGGATGATTTAATTTT |
