# Supplementary material for: Genomic Structure of and Genome-Wide Recombination in the Saccharomyces cerevisiae S288C Progenitor Isolate EM93
Source: PLoS One. 2011 Sep 26;6(9):e25211. doi: 10.1371/journal.pone.0025211 (PMC3180460; doi:10.1371/journal.pone.0025211)
Supplement: Table S2 — List of LSPs in EM93. Large sequence polymorphisms (LSPs; ≥500 bp) were identified based on low levels of the hybridization intensities of overlapping oligonucleotide features using the Integrated Genome Browser software (Affymetrix). A complete list of positions of identified LSPs is shown above. The association of LSPs with Ty3, Ty4, other Ty/LTR elements (potentially (Ty1), (Ty2) or (LTR) present in S288C), sub-telomeric regions (ST), S. paradoxus homology (S.p), or non-sub-telomeric (non-ST; all Ty and LTR associated LSPs are also non-ST) is also indicated above (Assoc. with). * (KIN3, CDC15, YAR019W-A, ARS110, PAU7, YAR023C, SUP56, tS(AGA)A, YARWsigma1, YARWdelta6, UIP3, YAR028W, YAR029W, PRM9, and MST29). (DOC) [file pone.0025211.s010.doc]

**TABLE S2**

**Large sequence polymorphisms** in EM93

| **LSP#** | | **Polymorphic and/or** | | **Chr.** | **Chr.** | **Chr. Position** | | **Presence in EM93-segregant** | | | | | | | |
| --- | --- | --- | --- | --- | --- | --- | --- | --- | --- | --- | --- | --- | --- | --- | --- |
|  |  | **deleted gene(s)** | | **feature** | **#** | **Start** | **Stop** | **2A** | **2B** | **2C** | **2D** | **3A** | **3B** | **3C** | **3D** |
| 1 |  | | *SEO1, ARS103, YAL066W* | ST | I | 2,828 | 11,644 | 0 | 0 | 0 | 0 | 0 | 1 | 0 | 0 |
| 2 |  | | * | *S.p* | I | 179,666 | 190,181 | 0 | 0 | 1 | 1 | 0 | 1 | 1 | 0 |
| 3 |  | | *YAR047C* | non-SP | I | 198,866 | 202,777 | 1 | 1 | 0 | 0 | 1 | 1 | 0 | 0 |
| 4 |  | | *IMD1, YAR075W* | ST | I | 226,886 | 230,075 | 1 | 1 | 0 | 0 | 1 | 1 | 0 | 0 |
| 5 |  | | - | (Ty1) | II | 265,501 | 266,187 | 0 | 0 | 0 | 0 | 0 | 1 | 1 | 0 |
| 6 |  | | - | (LTR) | III | 148,657 | 151,244 | 0 | 0 | 1 | 1 | 1 | 0 | 1 | 0 |
| 7 |  | | *ADH7, RDS1, AAD3* | ST | III | 308,225 | 315,601 | 0 | 1 | 1 | 0 | 0 | 1 | 0 | 1 |
| 8 |  | | *ARS504* | ST | V | 7,823 | 10,107 | 0 | 1 | 0 | 1 | 0 | 1 | 1 | 0 |
| 9 |  | | *AAD6 (3´)* | ST | VI | 15,320 | 16,784 | 0 | 0 | 1 | 1 | 1 | 0 | 0 | 1 |
| 10 |  | | - | (Ty2) | VI | 143,952 | 144,847 | 0 | 0 | 1 | 1 | 1 | 0 | 0 | 1 |
| 11 |  | | *ALD1 (3´)* | non-SP | VI | 205,005 | 205,914 | 0 | 0 | 1 | 1 | 1 | 0 | 0 | 1 |
| 12 |  | | *PRM8, MST27, YGL052W* | (LTR) | VII | 402,339 | 404,657 | 0 | 0 | 1 | 0 | 0 | 0 | 0 | 0 |
| 13 |  | | *TY3* | Ty3 | VII | 707,609 | 712,241 | 0 | 0 | 1 | 1 | 0 | 1 | 0 | 1 |
| 14 |  | | *MAL13, MAL11* | ST | VII | 1,069,041 | 1,076,119 | 1 | 1 | 1 | 1 | 1 | 1 | 1 | 1 |
| 15 |  | | *TY4* | Ty4 | VIII | 85,915 | 91,934 | 0 | 0 | 0 | 1 | 0 | 0 | 0 | 0 |
| 16 |  | | *YHL008C* | (LTR) | VIII | 93,293 | 94,934 | 0 | 0 | 1 | 1 | 1 | 0 | 0 | 1 |
| 17 |  | | *TY3* | Ty3 | IX | 205,644 | 210,309 | 0 | 0 | 1 | 1 | 0 | 1 | 0 | 1 |
| 18 |  | | *YIL029C* | (LTR) | IX | 300,709 | 301,477 | 1 | 1 | 0 | 0 | 0 | 1 | 1 | 0 |
| 19 |  | | *YIL014C-A* | (LTR) | IX | 325,121 | 325,713 | 0 | 0 | 1 | 1 | 1 | 0 | 0 | 1 |
| 20 |  | | *YIR042C* | ST | IX | 434,376 | 437,036 | 0 | 0 | 1 | 1 | 1 | 0 | 0 | 1 |
| 21 |  | | *YJL218W, REE1, IMA5* | ST | X | 21,255 | 25,375 | 0 | 0 | 1 | 1 | 1 | 1 | 0 | 0 |
| 22 |  | | *TY4* | Ty4 | X | 197,837 | 203,309 | 0 | 0 | 0 | 1 | 0 | 0 | 0 | 0 |
| 23 |  | | *AIF1, COS10* | ST | XIV | 777,020 | 781,260 | 0 | 1 | 0 | 1 | 0 | 1 | 1 | 0 |
| 24 |  | | *AAD15, BDS1, YOL163W, YOL162W* | ST | XV | 1,118 | 11,029 | 0 | 1 | 0 | 1 | 1 | 0 | 1 | 0 |
| 25 |  | | intergenic | ST | XV | 1,073,295 | 1,074,365 | 0 | 1 | 0 | 1 | 0 | 0 | 1 | 0 |
| 26 |  | | *(5´)YPL277C* | ST | XVI | 16,737 | 17,512 | 1 | 0 | 1 | 0 | 1 | 1 | 0 | 0 |
| 27 |  | | *TY4* | Ty4 | XVI | 437,284 | 442,703 | 0 | 0 | 0 | 1 | 0 | 0 | 0 | 0 |

Large sequence polymorphisms (LSPs; ≥ 500 bp) were identified based on low levels of the hybridization intensities of overlapping oligonucleotide features using the Integrated Genome Browser software (Affymetrix). A complete list of positions of identified LSPs is shown above. The association of LSPs with Ty3, Ty4, other Ty/LTR elements (potentially (Ty1), (Ty2) or (LTR) present in S288c), sub-telomeric regions (ST), *S. paradoxus* homology (*S.p*), or non-sub-telomeric (non-ST; all Ty and LTR associated LSPs are also non-ST) is also indicated above (Assoc. with). * (*KIN3, CDC15, YAR019W-A, ARS110, PAU7, YAR023C, SUP56, tS(AGA)A, YARWsigma1, YARWdelta6, UIP3, YAR028W, YAR029W, PRM9,* and *MST29*)
