# Supplementary material for: Genomic Structure of and Genome-Wide Recombination in the Saccharomyces cerevisiae S288C Progenitor Isolate EM93
Source: PLoS One. 2011 Sep 26;6(9):e25211. doi: 10.1371/journal.pone.0025211 (PMC3180460; doi:10.1371/journal.pone.0025211)
Supplement: Table S3 — Tiling Array probes that segregate 2∶2 in EM93. (DOC) [file pone.0025211.s011.doc]

**TABLE S3**

Tiling Array probes that segregate 2:2 in EM93

|  | **Total^1^** | **Tetrad 1** | **Tetrad 2** |
| --- | --- | --- | --- |
| **Chr.** | **# Probes** | **# 2:2 probes** | **# 2:2 probes** |
| I | 41,436 | 3,401 | 3,373 |
| II | 170,119 | 748 | 4,031 |
| III | 63,927 | 2,333 | 2,245 |
| IV | 624,858 | 5,039 | 5,026 |
| V | 118,808 | 2,291 | 2,245 |
| VI | 54,768 | 1,774 | 2,139 |
| VII | 225,318 | 4,117 | 4,028 |
| VIII | 111,848 | 1,990 | 1,868 |
| IX | 88,944 | 2,690 | 2,857 |
| X | 151,020 | 3,191 | 3,186 |
| XI | 143,000 | 3,050 | 3,200 |
| XII | 214,231 | 1,350 | 1,048 |
| XIII | 192,873 | 2,749 | 2,756 |
| XIV | 161,499 | 3,489 | 3,261 |
| XV | 226,315 | 7,159 | 6,591 |
| XVI | 194,285 | 2,771 | 2,698 |
| **Total #:** | **2,783,249** | **48,142** | **50,552** |

^1^Probes with unique hits in the S288C genome
