# Supplementary material for: Genomic Structure of and Genome-Wide Recombination in the Saccharomyces cerevisiae S288C Progenitor Isolate EM93
Source: PLoS One. 2011 Sep 26;6(9):e25211. doi: 10.1371/journal.pone.0025211 (PMC3180460; doi:10.1371/journal.pone.0025211)
Supplement: Table S4 — Summary of EM93 genotyping markers. (DOC) [file pone.0025211.s012.doc]

**TABLE S4**

**Summary of EM93 genotyping markers**

| **Chr.** | **Chr. length** | **# of Markers** | **Start** | **End** | **Coverage** | **Avg. Dist.** |
| --- | --- | --- | --- | --- | --- | --- |
| I | 230,208 | 74 | 407 | 230,057 | 99.8% | 3.1 |
| II | 813,178 | 160 | 6,027 | 809,928 | 98.9% | 5.0 |
| III | 316,617 | 52 | 108,780 | 313,055 | 64.5% | 3.9 |
| IV | 1,531,919 | 272 | 2,561 | 746,159 | 48.5% | 2.7 |
| V | 576,869 | 98 | 4,078 | 368,996 | 63.3% | 3.7 |
| VI | 270,148 | 71 | 8,181 | 258,527 | 92.7% | 3.5 |
| VII | 1,090,947 | 179 | 2,265 | 808,358 | 73.9% | 4.5 |
| VIII | 562,643 | 159 | 58,658 | 556,477 | 88.5% | 3.1 |
| IX | 439,885 | 117 | 45,971 | 439,767 | 89.5% | 3.4 |
| X | 745,741 | 161 | 19,570 | 743,107 | 97.0% | 4.5 |
| XI | 666,454 | 250 | 3,015 | 665,018 | 99.3% | 2.6 |
| XII | 1,078,175 | 119 | 14,023 | 445,168 | 40.0% | 3.6 |
| XIII | 924,429 | 282 | 26,807 | 924,242 | 97.1% | 3.1 |
| XIV | 784,334 | 291 | 17,680 | 781,233 | 97.4% | 2.6 |
| XV | 1,091,289 | 459 | 193 | 1,071,269 | 98.1% | 2.3 |
| XVI | 948,062 | 221 | 12,978 | 730,923 | 75.7% | 3.3 |
| **Total #:** |  | **2,965** |  |  | **Avg. 76.5%** | **Avg. 3.4 kb** |
