# Supplementary material for: Genomic Structure of and Genome-Wide Recombination in the Saccharomyces cerevisiae S288C Progenitor Isolate EM93
Source: PLoS One. 2011 Sep 26;6(9):e25211. doi: 10.1371/journal.pone.0025211 (PMC3180460; doi:10.1371/journal.pone.0025211)
Supplement: Table S5 — Crossover interference on chromosome XI. (DOC) [file pone.0025211.s013.doc]

**TABLE S5**

Crossover interference on chromosome XI

| **Interval 1¹** | | | **Interval 2¹** | | |  | **Interval 1**  **Total** | **Interval 2**  **CO No-CO** | | ***p-value*^2^** |
| --- | --- | --- | --- | --- | --- | --- | --- | --- | --- | --- |
| 3,015 | - | 44,241 | 44,241 | - | 97,884 | CO | 37 | 9 | 28 |  |
|  |  |  |  |  |  | No-CO | 83 | 51 | 32 | **<0.001** |
|  |  |  |  |  |  |  |  |  |  |  |
| 44,241 | - | 97,884 | 97,884 | - | 145,614 | CO | 60 | 6 | 54 |  |
|  |  |  |  |  |  | No-CO | 60 | 16 | 44 | 0.016 |
|  |  |  |  |  |  |  |  |  |  |  |
| 97,884 | - | 145,614 | 145,614 | - | 199,531 | CO | 22 | 9 | 13 |  |
|  |  |  |  |  |  | No-CO | 98 | 28 | 70 | 0.189 |
|  |  |  |  |  |  |  |  |  |  |  |
| 145,614 | - | 199,531 | 199,531 | - | 249,938 | CO | 37 | 4 | 33 |  |
|  |  |  |  |  |  | No-CO | 83 | 35 | 48 | **<0.001** |
|  |  |  |  |  |  |  |  |  |  |  |
| 199,531 | - | 249,938 | 249,938 | - | 301,148 | CO | 39 | 13 | 26 |  |
|  |  |  |  |  |  | No-CO | 81 | 42 | 39 | 0.043 |
|  |  |  |  |  |  |  |  |  |  |  |
| 249,938 | - | 301,148 | 301,148 | - | 350,329 | CO | 55 | 9 | 46 |  |
|  |  |  |  |  |  | No-CO | 65 | 20 | 45 | **0.004** |
|  |  |  |  |  |  |  |  |  |  |  |
| 301,148 | - | 350,329 | 350,329 | - | 410,63 | CO | 29 | 5 | 24 |  |
|  |  |  |  |  |  | No-CO | 91 | 48 | 43 | **<0.001** |
|  |  |  |  |  |  |  |  |  |  |  |
| 350,329 | - | 410,63 | 410,63 | - | 462,124 | CO | 53 | 4 | 49 |  |
|  |  |  |  |  |  | No-CO | 67 | 17 | 50 | 0.009 |
|  |  |  |  |  |  |  |  |  |  |  |
| 410,63 | - | 462,124 | 462,124 | - | 527,218 | CO | 21 | 6 | 15 |  |
|  |  |  |  |  |  | No-CO | 99 | 51 | 48 | 0.046 |
|  |  |  |  |  |  |  |  |  |  |  |
| 462,124 | - | 527,218 | 527,218 | - | 570,572 | CO | 57 | 11 | 46 |  |
|  |  |  |  |  |  | No-CO | 63 | 21 | 42 | 0.062 |
|  |  |  |  |  |  |  |  |  |  |  |
| 527,218 | - | 570,572 | 570,572 | - | 614,129 | CO | 32 | 8 | 24 |  |
|  |  |  |  |  |  | No-CO | 88 | 27 | 61 | 0.358 |
|  |  |  |  |  |  |  |  |  |  |  |
| 570,572 | - | 614,129 | 614,129 | - | 665,018 | CO | 35 | 11 | 24 |  |
|  |  |  |  |  |  | No-CO | 85 | 39 | 46 | 0.104 |

¹ Intervals are shown in kilobases.

²Calculated *p*-value using Fisher exact test, two sided *p-*value and corrected for multiple

comparisons (Bonferroni), bold *p-value* <0.0042.
