# Supplementary material for: Genomic Structure of and Genome-Wide Recombination in the Saccharomyces cerevisiae S288C Progenitor Isolate EM93
Source: PLoS One. 2011 Sep 26;6(9):e25211. doi: 10.1371/journal.pone.0025211 (PMC3180460; doi:10.1371/journal.pone.0025211)
Supplement: Table S6 — Crossover interference on chromosome XV. (DOC) [file pone.0025211.s014.doc]

**TABLE S6**

Crossover interference on chromosome XV

|  | | | |  | | |  | **Interval 1** | **Interval 2** | |  |
| --- | --- | --- | --- | --- | --- | --- | --- | --- | --- | --- | --- |
| **Interval 1¹** | | | | **Interval 2¹** | | |  | **Total** | **CO** | **No-CO** | ***P*-value^2^** |
| 0.2 | - | 39.4 | 39.4 | | - | 80.4 | CO | 20 | 4 | 16 | 0.19 |
|  |  |  | |  |  |  | No-CO | 100 | 37 | 63 |  |
| 39.4 | - | 80.4 | | 80.4 | - | 123.7 | CO | 41 | 3 | 38 | 0.026 |
|  |  |  | |  |  |  | No-CO | 79 | 19 | 60 |  |
| 80.4 | - | 123.7 | | 123.7 | - | 177.7 | CO | 22 | 7 | 15 | 1.00 |
|  |  |  | |  |  |  | No-CO | 98 | 32 | 66 |  |
| 123.7 | - | 177.7 | | 177.7 | - | 230.8 | CO | 39 | 16 | 23 | 0.12 |
|  |  |  | |  |  |  | No-CO | 81 | 46 | 35 |  |
| 177.7 | - | 230.8 | | 230.8 | - | 274.5 | CO | 62 | 17 | 45 | 0.009 |
|  |  |  | |  |  |  | No-CO | 58 | 30 | 28 |  |
| 230.8 | - | 274.5 | | 274.5 | - | 315.7 | CO | 47 | 6 | 41 | 0.01 |
|  |  |  | |  |  |  | No-CO | 73 | 25 | 48 |  |
| 274.5 | - | 315.7 | | 315.7 | - | 367.2 | CO | 31 | 0 | 31 | 0.33 |
|  |  |  | |  |  |  | No-CO | 89 | 5 | 84 |  |
| 315.7 | - | 367.2 | | 367.2 | - | 423.8 | CO | 5 | 1 | 4 | 0.66 |
|  |  |  | |  |  |  | No-CO | 115 | 39 | 76 |  |
| 367.2 | - | 423.8 | | 423.8 | - | 475.7 | CO | 40 | 7 | 33 | **>0.001** |
|  |  |  | |  |  |  | No-CO | 80 | 44 | 36 |  |
| 423.8 | - | 475.7 | | 475.7 | - | 520.3 | CO | 51 | 9 | 42 | 0.009 |
|  |  |  | |  |  |  | No-CO | 69 | 28 | 41 |  |
| 475.7 | - | 520.3 | | 520.3 | - | 587.8 | CO | 37 | 11 | 26 | 0.15 |
|  |  |  | |  |  |  | No-CO | 83 | 37 | 46 |  |
| 520.3 | - | 587.8 | | 587.8 | - | 646.8 | CO | 48 | 14 | 34 | 0.55 |
|  |  |  | |  |  |  | No-CO | 72 | 26 | 46 |  |
| 587.8 | - | 646.8 | | 646.8 | - | 703.1 | CO | 40 | 10 | 30 | 0.011 |
|  |  |  | |  |  |  | No-CO | 80 | 40 | 40 |  |
| 646.8 | - | 703.1 | | 703.1 | - | 751.3 | CO | 50 | 11 | 39 | 0.40 |
|  |  |  | |  |  |  | No-CO | 70 | 21 | 49 |  |
| 703.1 | - | 751.3 | | 751.3 | - | 780.7 | CO | 32 | 3 | 29 | 0.55 |
|  |  |  | |  |  |  | No-CO | 88 | 14 | 74 |  |
| 751.3 | - | 780.7 | | 780.7 | - | 830.8 | CO | 17 | 5 | 12 | 0.42 |
|  |  |  | |  |  |  | No-CO | 103 | 43 | 60 |  |
| 780.7 | - | 830.8 | | 830.8 | - | 880.8 | CO | 48 | 7 | 41 | **0.002** |
|  |  |  | |  |  |  | No-CO | 72 | 34 | 38 |  |
| 830.8 | - | 880.8 | | 880.8 | - | 945.6 | CO | 41 | 23 | 18 | 0.32 |
|  |  |  | |  |  |  | No-CO | 79 | 52 | 27 |  |
| 880.8 | - | 945.6 | | 945.6 | - | 996.7 | CO | 75 | 20 | 55 | 0.31 |
|  |  |  | |  |  |  | No-CO | 45 | 16 | 29 |  |
| 945.6 | - | 996.7 | | 996.7 | - | 1071.3 | CO | 36 | 14 | 22 | 0.11 |
|  |  |  | |  |  |  | No-CO | 84 | 47 | 37 |  |

¹ Intervals are shown in kilobases.

²Calculated *p*-value using Fisher exact test, two sided *p-*value and corrected for multiple

comparisons (Bonferroni), bold *p-value* <0.0025.
