# Supplementary material for: Genomic Structure of and Genome-Wide Recombination in the Saccharomyces cerevisiae S288C Progenitor Isolate EM93
Source: PLoS One. 2011 Sep 26;6(9):e25211. doi: 10.1371/journal.pone.0025211 (PMC3180460; doi:10.1371/journal.pone.0025211)
Supplement: Table S7 — Location of Hotspots. (DOC) [file pone.0025211.s015.doc]

**TABLE S7**

Locations of EM93 Hotspots

| Chr. | HS# | #CI | Interval | | | CO | NCOGC |
| --- | --- | --- | --- | --- | --- | --- | --- |
| 1 | 1 | 1 | 28109 | - | 29411 | 0,0 | 4,0 |
| 1 | 2 | 1 | 31156 | - | 34966 | 2,8 | 1,8 |
| 1 | 3 | 2 | 59341 | - | 80290 | 3,4 | 0,3 |
| 1 |  | 2 | 80290 | - | 81436 | 1,9 | 4,6 |
| 1 | 4 | 1 | 121480 | - | 135321 | 2,7 | 0,4 |
| 1 | 5 | 2 | 189875 | - | 196098 | 5,9 | 2,5 |
| 1 |  | 2 | 196098 | - | 198648 | 3,0 | 1,4 |
| 1 | 6 | 1 | 202092 | - | 207154 | 0,9 | 8,3 |
|  |  |  |  |  |  |  |  |
| Chr. | HS# | #CI | Interval | | | CO | NCOGC |
| 2 | 7 | 1 | 40974 | - | 46708 | 2,5 | 2,7 |
| 2 | 8 | 2 | 150186 | - | 158790 | 2,3 | 1,2 |
| 2 |  | 2 | 158790 | - | 162250 | 3,4 | 0,0 |
| 2 | 9 | 1 | 190968 | - | 193537 | 4,2 | 6,1 |
| 2 | 10 | 2 | 280672 | - | 290464 | 2,4 | 1,2 |
| 2 |  | 2 | 290464 | - | 302383 | 2,4 | 0,1 |
| 2 | 11 | 1 | 389572 | - | 394406 | 2,7 | 0,0 |
| 2 | 12 | 1 | 407545 | - | 410961 | 2,2 | 2,0 |
| 2 | 13 | 1 | 584373 | - | 586941 | 0,4 | 2,7 |
| 2 | 14 | 1 | 649572 | - | 655436 | 2,4 | 0,3 |
| 2 | 15 | 1 | 660217 | - | 664704 | 2,4 | 0,4 |
| 2 | 16 | 1 | 699855 | - | 701859 | 4,9 | 5,2 |
| 2 | 17 | 2 | 730841 | - | 740339 | 2,3 | 0,0 |
| 2 |  | 2 | 740339 | - | 745172 | 2,7 | 1,1 |
| 2 | 18 | 1 | 775543 | - | 777859 | 3,3 | 0,8 |
| 2 | 19 | 1 | 782358 | - | 784788 | 2,2 | 0,0 |
| 2 | 20 | 1 | 797323 | - | 799494 | 2,5 | 0,8 |
|  |  |  |  |  |  |  |  |
| Chr. | HS# | #CI | Interval | | | CO | NCOGC |
| 3 | 21 | 1 | 156945 | - | 172800 | 3,1 | 1,2 |
| 3 | 22 | 1 | 208068 | - | 227750 | 3,0 | 0,1 |
| 3 | 23 | 1 | 243767 | - | 246792 | 3,6 | 4,6 |
| 3 | 24 | 1 | 259703 | - | 262947 | 2,3 | 1,1 |
|  |  |  |  |  |  |  |  |
| Chr. | HS# | #CI | Interval | | | CO | NCOGC |
| 4 | 25 | 2 | 85329 | - | 89535 | 0,3 | 3,3 |
| 4 |  | 2 | 89535 | - | 92660 | 4,4 | 0,6 |
| 4 | 26 | 1 | 102688 | - | 106130 | 0,0 | 3,0 |
| 4 | 27 | 1 | 156303 | - | 160999 | 3,0 | 0,4 |
| 4 | 28 | 1 | 163885 | - | 167285 | 0,6 | 2,1 |
| 4 | 29 | 1 | 189496 | - | 193103 | 0,3 | 2,4 |
| 4 | 30 | 1 | 196439 | - | 199730 | 4,5 | 3,2 |
| 4 | 31 | 1 | 263805 | - | 267349 | 0,9 | 2,5 |
| 4 | 32 | 1 | 278681 | - | 282839 | 2,3 | 0,0 |
| 4 | 33 | 1 | 331896 | - | 334879 | 1,4 | 2,3 |
| 4 | 34 | 1 | 342616 | - | 345748 | 0,0 | 2,2 |
| 4 | 35 | 1 | 355002 | - | 358542 | 1,2 | 2,5 |
| 4 | 36 | 1 | 397846 | - | 400883 | 0,0 | 3,4 |
| 4 | 37 | 1 | 521459 | - | 551575 | 2,6 | 0,3 |
| 4 | 38 | 1 | 691737 | - | 694225 | 0,4 | 2,1 |
| 4 | 39 | 1 | 698250 | - | 701438 | 2,3 | 0,5 |
| 4 | 40 | 1 | 713092 | - | 715612 | 0,0 | 2,8 |
|  |  |  |  |  |  |  |  |
| Chr. | HS# | #CI | Interval | | | CO | NCOGC |
| 5 | 41 | 1 | 13307 | - | 15466 | 1,0 | 4,0 |
| 5 | 42 | 1 | 24894 | - | 29249 | 2,5 | 6,0 |
| 5 | 43 | 2 | 39169 | - | 43058 | 2,2 | 3,1 |
| 5 |  | 2 | 43058 | - | 46317 | 1,0 | 2,7 |
| 5 | 44 | 1 | 63348 | - | 73653 | 2,6 | 0,0 |
| 5 | 45 | 1 | 216708 | - | 219261 | 0,4 | 2,0 |
| 5 | 46 | 1 | 223145 | - | 226846 | 3,2 | 0,0 |
|  |  |  |  |  |  |  |  |
| Chr. | HS# | #CI | Interval | | | CO | NCOGC |
| 6 | 47 | 2 | 31513 | - | 35896 | 0,7 | 4,8 |
| 6 |  | 2 | 35896 | - | 40318 | 2,4 | 0,4 |
| 6 | 48 | 1 | 63001 | - | 68726 | 3,6 | 0,6 |
| 6 | 49 | 1 | 73217 | - | 79741 | 2,2 | 0,5 |
| 6 | 50 | 1 | 89159 | - | 98261 | 3,6 | 0,6 |
| 6 | 51 | 1 | 167242 | - | 181203 | 2,7 | 0,4 |
| 6 | 52 | 3 | 207830 | - | 226159 | 2,9 | 1,0 |
| 6 |  | 3 | 226159 | - | 228481 | 1,9 | 3,8 |
| 6 |  | 3 | 228481 | - | 231617 | 1,4 | 5,6 |
|  |  |  |  |  |  |  |  |
| Chr. | HS# | #CI | Interval | | | CO | NCOGC |
| 7 | 53 | 2 | 19180 | - | 22000 | 0,8 | 3,7 |
| 7 |  | 2 | 22000 | - | 29347 | 4,1 | 0,5 |
| 7 | 54 | 1 | 165776 | - | 171277 | 0,4 | 2,9 |
| 7 | 55 | 1 | 182390 | - | 185982 | 3,0 | 2,4 |
| 7 | 56 | 1 | 193458 | - | 197936 | 1,0 | 2,3 |
| 7 | 57 | 1 | 200555 | - | 204928 | 0,2 | 2,4 |
| 7 | 58 | 1 | 214751 | - | 217443 | 0,0 | 2,6 |
| 7 | 59 | 2 | 223262 | - | 230207 | 2,6 | 1,0 |
| 7 |  | 2 | 230207 | - | 232187 | 1,1 | 4,4 |
| 7 | 60 | 1 | 286692 | - | 293241 | 0,2 | 2,1 |
| 7 | 61 | 1 | 304210 | - | 307048 | 2,3 | 1,8 |
| 7 | 62 | 1 | 352535 | - | 355688 | 0,3 | 2,2 |
| 7 | 63 | 1 | 390473 | - | 402842 | 2,4 | 0,0 |
| 7 | 64 | 1 | 405404 | - | 408707 | 0,3 | 3,7 |
| 7 | 65 | 1 | 675388 | - | 677703 | 4,2 | 3,8 |
| 7 | 66 | 1 | 730219 | - | 734615 | 1,0 | 4,4 |
| 7 | 67 | 1 | 779832 | - | 783979 | 2,9 | 0,4 |
|  |  |  |  |  |  |  |  |
| Chr. | HS# | #CI | Interval | | | CO | NCOGC |
| 8 | 68 | 1 | 64340 | - | 73677 | 2,4 | 0,4 |
| 8 | 69 | 1 | 95308 | - | 98503 | 1,0 | 2,7 |
| 8 | 70 | 1 | 121713 | - | 123594 | 3,5 | 8,3 |
| 8 | 71 | 2 | 139384 | - | 141666 | 1,4 | 6,1 |
| 8 |  | 2 | 141666 | - | 146647 | 5,2 | 1,1 |
| 8 | 72 | 2 | 183892 | - | 188828 | 2,4 | 3,9 |
| 8 |  | 2 | 188828 | - | 192725 | 3,9 | 0,9 |
| 8 | 73 | 1 | 214235 | - | 216321 | 0,0 | 4,2 |
| 8 | 74 | 1 | 225208 | - | 227205 | 0,0 | 3,5 |
| 8 | 75 | 2 | 231143 | - | 233534 | 3,2 | 0,7 |
| 8 |  | 2 | 233534 | - | 236804 | 1,3 | 2,7 |
| 8 | 76 | 1 | 251782 | - | 255639 | 0,3 | 2,3 |
| 8 | 77 | 1 | 278192 | - | 279878 | 0,6 | 3,1 |
| 8 | 78 | 1 | 286229 | - | 289016 | 0,8 | 4,4 |
| 8 | 79 | 1 | 299716 | - | 302221 | 0,0 | 4,2 |
| 8 | 80 | 1 | 393005 | - | 396986 | 0,0 | 2,6 |
| 8 | 81 | 1 | 450755 | - | 466286 | 2,4 | 0,2 |
|  |  |  |  |  |  |  |  |
| Chr. | HS# | #CI | Interval | | | CO | NCOGC |
| 9 | 82 | 2 | 45971 | - | 47640 | 1,9 | 8,4 |
| 9 |  | 2 | 47640 | - | 55057 | 3,4 | 2,8 |
| 9 | 83 | 1 | 82604 | - | 85662 | 1,8 | 2,9 |
| 9 | 84 | 2 | 89149 | - | 96848 | 1,3 | 2,0 |
| 9 |  | 2 | 96848 | - | 99744 | 3,0 | 0,6 |
| 9 | 85 | 1 | 158897 | - | 163061 | 2,3 | 2,9 |
| 9 | 86 | 1 | 164868 | - | 167620 | 0,4 | 6,3 |
| 9 | 87 | 2 | 171498 | - | 174672 | 1,4 | 2,7 |
| 9 |  | 2 | 174672 | - | 181412 | 2,4 | 1,0 |
| 9 | 88 | 2 | 260006 | - | 266704 | 1,0 | 2,3 |
| 9 |  | 2 | 266704 | - | 272654 | 2,0 | 2,6 |
| 9 | 89 | 1 | 308351 | - | 310512 | 1,0 | 2,4 |
| 9 | 90 | 2 | 331648 | - | 335012 | 0,3 | 2,1 |
| 9 |  | 2 | 335012 | - | 338027 | 1,1 | 2,3 |
| 9 | 91 | 2 | 375196 | - | 378374 | 1,0 | 2,2 |
| 9 |  | 2 | 378374 | - | 386794 | 3,0 | 0,8 |
| 9 | 92 | 1 | 396287 | - | 399362 | 0,7 | 3,4 |
|  |  |  |  |  |  |  |  |
| Chr. | HS# | #CI | Interval | | | CO | NCOGC |
| 10 | 93 | 1 | 27189 | - | 31600 | 1,0 | 2,4 |
| 10 | 94 | 1 | 50993 | - | 54943 | 0,3 | 2,6 |
| 10 | 95 | 1 | 69361 | - | 73698 | 0,5 | 2,0 |
| 10 | 96 | 2 | 184143 | - | 188446 | 1,3 | 2,8 |
| 10 |  | 2 | 188446 | - | 191363 | 3,7 | 2,4 |
| 10 | 97 | 1 | 277666 | - | 284585 | 2,2 | 0,3 |
| 10 | 98 | 1 | 292834 | - | 298812 | 2,2 | 0,3 |
| 10 | 99 | 1 | 311970 | - | 316435 | 2,9 | 4,7 |
| 10 | 100 | 1 | 319675 | - | 325700 | 0,2 | 2,3 |
| 10 | 101 | 1 | 682296 | - | 686395 | 1,3 | 2,6 |
| 10 | 102 | 1 | 697785 | - | 705481 | 2,0 | 2,7 |
| 10 | 103 | 1 | 712863 | - | 727973 | 2,6 | 0,1 |
|  |  |  |  |  |  |  |  |
| Chr. | HS# | #CI | Interval | | | CO | NCOGC |
| 11 | 104 | 3 | 16402 | - | 19264 | 0,0 | 3,7 |
| 11 |  | 3 | 19264 | - | 24230 | 1,5 | 3,5 |
| 11 |  | 3 | 24230 | - | 26695 | 1,3 | 2,1 |
| 11 | 105 | 1 | 35594 | - | 44241 | 2,3 | 0,0 |
| 11 | 106 | 1 | 64544 | - | 67628 | 1,1 | 3,4 |
| 11 | 107 | 1 | 74096 | - | 91778 | 2,1 | 0,3 |
| 11 | 108 | 1 | 96539 | - | 99632 | 0,3 | 4,5 |
| 11 | 109 | 1 | 115981 | - | 118301 | 0,5 | 4,5 |
| 11 | 110 | 1 | 165239 | - | 167774 | 4,3 | 5,5 |
| 11 | 111 | 1 | 221667 | - | 226592 | 0,9 | 2,1 |
| 11 | 112 | 1 | 246340 | - | 249487 | 0,0 | 2,2 |
| 11 | 113 | 1 | 274949 | - | 287471 | 2,7 | 0,0 |
| 11 | 114 | 1 | 306045 | - | 308387 | 2,3 | 3,7 |
| 11 | 115 | 1 | 312990 | - | 315478 | 0,0 | 2,1 |
| 11 | 116 | 1 | 398507 | - | 410473 | 2,2 | 0,0 |
| 11 | 117 | 1 | 488174 | - | 492956 | 0,7 | 4,7 |
| 11 | 118 | 1 | 522304 | - | 527218 | 3,5 | 2,5 |
| 11 | 119 | 1 | 578505 | - | 581569 | 0,4 | 6,8 |
| 11 | 120 | 1 | 611772 | - | 614129 | 1,8 | 3,7 |
| 11 | 121 | 2 | 627008 | - | 630662 | 4,1 | 4,8 |
| 11 |  | 2 | 630662 | - | 640437 | 2,4 | 0,0 |
| 11 | 122 | 1 | 655763 | - | 657803 | 0,0 | 2,6 |
|  |  |  |  |  |  |  |  |
| Chr. | HS# | #CI | Interval | | | CO | NCOGC |
| 12 | 123 | 1 | 17856 | - | 24299 | 3,4 | 0,0 |
| 12 | 124 | 1 | 44670 | - | 48816 | 1,3 | 3,8 |
| 12 | 125 | 1 | 51935 | - | 54346 | 0,0 | 4,3 |
| 12 | 126 | 2 | 111811 | - | 113963 | 0,5 | 3,2 |
| 12 |  | 2 | 113963 | - | 126008 | 2,3 | 0,4 |
| 12 | 127 | 1 | 250573 | - | 273530 | 3,1 | 0,0 |
| 12 | 128 | 1 | 296456 | - | 301274 | 0,0 | 2,5 |
|  |  |  |  |  |  |  |  |
| Chr. | HS# | #CI | Interval | | | CO | NCOGC |
| 13 | 129 | 1 | 26807 | - | 28323 | 1,4 | 2,3 |
| 13 | 130 | 2 | 34788 | - | 37826 | 0,7 | 2,3 |
| 13 |  | 2 | 37826 | - | 40359 | 3,0 | 0,7 |
| 13 | 131 | 1 | 118664 | - | 121926 | 0,0 | 2,1 |
| 13 | 132 | 1 | 137462 | - | 142173 | 0,9 | 2,6 |
| 13 | 133 | 1 | 227950 | - | 230925 | 0,7 | 4,1 |
| 13 | 134 | 1 | 242842 | - | 251591 | 3,3 | 0,2 |
| 13 | 135 | 1 | 299927 | - | 301978 | 2,6 | 0,9 |
| 13 | 136 | 1 | 344461 | - | 356987 | 3,0 | 0,0 |
| 13 | 137 | 1 | 651366 | - | 653908 | 0,9 | 2,1 |
| 13 | 138 | 2 | 693734 | - | 695272 | 0,0 | 2,3 |
| 13 |  | 2 | 695272 | - | 703848 | 3,2 | 0,2 |
| 13 | 139 | 1 | 712374 | - | 723703 | 2,2 | 0,0 |
| 13 | 140 | 1 | 774672 | - | 777820 | 0,7 | 3,3 |
| 13 | 141 | 1 | 791962 | - | 796868 | 3,8 | 4,3 |
| 13 | 142 | 2 | 850644 | - | 853786 | 2,8 | 2,2 |
| 13 |  | 2 | 853786 | - | 863526 | 3,8 | 1,1 |
| 13 | 143 | 2 | 908605 | - | 912197 | 0,0 | 2,4 |
| 13 |  | 2 | 912197 | - | 916623 | 2,2 | 1,2 |
|  |  |  |  |  |  |  |  |
| Chr. | HS# | #CI | Interval | | | CO | NCOGC |
| 14 | 144 | 1 | 55889 | - | 62820 | 2,2 | 1,3 |
| 14 | 145 | 2 | 72709 | - | 77011 | 0,8 | 3,2 |
| 14 |  | 2 | 77011 | - | 79941 | 0,4 | 2,4 |
| 14 | 146 | 1 | 102532 | - | 106861 | 0,3 | 2,0 |
| 14 | 147 | 1 | 184130 | - | 190417 | 2,4 | 0,0 |
| 14 | 148 | 1 | 379684 | - | 382321 | 0,8 | 3,3 |
| 14 | 149 | 1 | 483721 | - | 493316 | 2,8 | 0,0 |
| 14 | 150 | 1 | 538220 | - | 544977 | 1,9 | 2,3 |
| 14 | 151 | 1 | 552240 | - | 554136 | 2,3 | 0,9 |
| 14 | 152 | 1 | 612511 | - | 614571 | 0,0 | 5,1 |
| 14 | 153 | 1 | 651836 | - | 655855 | 2,4 | 0,0 |
| 14 | 154 | 1 | 707390 | - | 710441 | 0,4 | 3,4 |
| 14 | 155 | 1 | 729478 | - | 732844 | 1,9 | 2,6 |
| 14 | 156 | 1 | 735876 | - | 737836 | 0,0 | 4,4 |
| 14 | 157 | 1 | 740580 | - | 742825 | 0,5 | 3,1 |
| 14 | 158 | 1 | 753303 | - | 755557 | 0,0 | 4,6 |
| 14 | 159 | 2 | 758677 | - | 760990 | 0,0 | 5,3 |
| 14 |  | 2 | 760990 | - | 768895 | 2,2 | 0,9 |
|  |  |  |  |  |  |  |  |
| Chr. | HS# | #CI | Interval | | | CO | NCOGC |
| 15 | 160 | 1 | 58148 | - | 62854 | 2,1 | 0,0 |
| 15 | 161 | 2 | 65807 | - | 71614 | 1,5 | 3,6 |
| 15 |  | 2 | 71614 | - | 74107 | 3,0 | 2,8 |
| 15 | 162 | 1 | 101963 | - | 104791 | 1,1 | 2,5 |
| 15 | 163 | 1 | 140477 | - | 146051 | 3,1 | 2,2 |
| 15 | 164 | 1 | 158373 | - | 160747 | 0,9 | 5,1 |
| 15 | 165 | 1 | 160747 | - | 163930 | 1,0 | 3,3 |
| 15 | 166 | 1 | 173898 | - | 177004 | 0,7 | 5,6 |
| 15 | 167 | 1 | 274542 | - | 276133 | 0,7 | 3,3 |
| 15 | 168 | 1 | 284509 | - | 289637 | 2,5 | 1,4 |
| 15 | 169 | 1 | 294535 | - | 296971 | 2,2 | 2,1 |
| 15 | 170 | 1 | 305521 | - | 309235 | 0,3 | 2,3 |
| 15 | 171 | 1 | 337453 | - | 343157 | 0,4 | 3,1 |
| 15 | 172 | 1 | 423580 | - | 431830 | 2,6 | 0,8 |
| 15 | 173 | 1 | 442770 | - | 449091 | 2,4 | 1,1 |
| 15 | 174 | 1 | 456970 | - | 461602 | 0,7 | 2,6 |
| 15 | 175 | 1 | 477670 | - | 482591 | 2,2 | 0,7 |
| 15 | 176 | 1 | 490932 | - | 495035 | 1,1 | 2,5 |
| 15 | 177 | 1 | 518788 | - | 521622 | 0,4 | 3,1 |
| 15 | 178 | 1 | 547011 | - | 553065 | 2,1 | 0,9 |
| 15 | 179 | 1 | 566414 | - | 569767 | 1,3 | 2,1 |
| 15 | 180 | 2 | 580190 | - | 586125 | 1,6 | 2,3 |
| 15 |  | 2 | 586125 | - | 589256 | 1,0 | 2,2 |
| 15 | 181 | 2 | 594437 | - | 601015 | 0,0 | 2,4 |
| 15 |  | 2 | 601015 | - | 606370 | 1,8 | 3,6 |
| 15 | 182 | 1 | 773949 | - | 776718 | 0,0 | 3,8 |
| 15 | 183 | 1 | 779083 | - | 782465 | 0,0 | 5,2 |
| 15 | 184 | 1 | 927497 | - | 945596 | 3,2 | 0,2 |
| 15 | 185 | 3 | 979956 | - | 983399 | 1,3 | 3,5 |
| 15 |  | 3 | 983399 | - | 985916 | 3,4 | 2,8 |
| 15 |  | 3 | 985916 | - | 989604 | 3,5 | 5,7 |
| 15 | 186 | 1 | 993738 | - | 996685 | 5,1 | 2,4 |
| 15 | 187 | 1 | 1040502 | - | 1047946 | 0,7 | 2,1 |
| 15 | 188 | 1 | 1063968 | - | 1065696 | 0,6 | 2,0 |
|  |  |  |  |  |  |  |  |
| Chr. | HS# | #CI | Interval | | | CO | NCOGC |
| 16 | 189 | 1 | 18930 | - | 21674 | 0,0 | 2,5 |
| 16 | 190 | 1 | 36965 | - | 40994 | 4,0 | 2,2 |
| 16 | 191 | 1 | 44104 | - | 47122 | 3,2 | 4,6 |
| 16 | 192 | 1 | 73066 | - | 79480 | 2,0 | 0,3 |
| 16 | 193 | 1 | 104267 | - | 108212 | 0,3 | 2,7 |
| 16 | 194 | 1 | 161450 | - | 170624 | 2,5 | 0,8 |
| 16 | 195 | 1 | 252603 | - | 258310 | 2,5 | 0,3 |
| 16 | 196 | 1 | 261895 | - | 263783 | 0,6 | 2,8 |
| 16 | 197 | 1 | 277415 | - | 283920 | 2,0 | 0,0 |
| 16 | 198 | 1 | 345802 | - | 347210 | 0,8 | 7,4 |
| 16 | 199 | 1 | 356783 | - | 359449 | 1,6 | 2,6 |
| 16 | 200 | 5 | 642443 | - | 644553 | 1,0 | 2,5 |
| 16 |  | 5 | 644553 | - | 649712 | 2,1 | 0,7 |
| 16 |  | 5 | 649712 | - | 651812 | 0,0 | 2,5 |
| 16 |  | 5 | 651812 | - | 654308 | 2,2 | 2,1 |
| 16 |  | 5 | 654308 | - | 657473 | 1,0 | 2,8 |
| 16 | 201 | 1 | 674526 | - | 679618 | 0,9 | 3,4 |
| 16 | 202 | 1 | 708843 | - | 711204 | 1,8 | 3,0 |
| 16 | 203 | 2 | 724984 | - | 726624 | 0,7 | 3,2 |
| 16 |  | 2 | 726624 | - | 730923 | 3,3 | 8,5 |

HS# = Hot spot number. #CI = number of consecutive intervals in hot spot (see Materials and Methods). Reciprocal Crossovers (CO) and Non-Crossover Gene conversion (NCOGC) events are presented by the fold change of events (number of events/expected number of events) in the indicated interval(s).
