# Supplementary material for: Genomic Structure of and Genome-Wide Recombination in the Saccharomyces cerevisiae S288C Progenitor Isolate EM93
Source: PLoS One. 2011 Sep 26;6(9):e25211. doi: 10.1371/journal.pone.0025211 (PMC3180460; doi:10.1371/journal.pone.0025211)
Supplement: Table S8 — Recombination in intervals adjacent to LSPs. (DOC) [file pone.0025211.s016.doc]

**TABLE S8**

Number of recombination events in intervals adjacent to LSPs

| LSP# | Assoc. With | Chr. # | Start | Stop | Markers/Regions^2^ | | | CO^3^ | NCOGC^3^ |
| --- | --- | --- | --- | --- | --- | --- | --- | --- | --- |
| 1 | ST | I | 2,828 | 11,644 | 407 | - | 3875 | 0.00 | 0.00 |
|  |  |  |  |  | 3875 | - | 6350 | 0.00 | 0.00 |
|  |  |  |  |  | 6350 | - | 7917 | 0.00 | 0.00 |
|  |  |  |  |  | 7917 | - | 10572 | 0.00 | 0.00 |
|  |  |  |  |  | 10572 | - | 12540 | 0.00 | 0.00 |
|  |  |  |  |  |  |  |  |  |  |
| 2 | *S.p* | I | 179,666 | 190,181 | 179902 | - | 182271 | 0.00 | 0.74 |
|  |  |  |  |  | 182271 | - | 183901 | 0.00 | 0.00 |
|  |  |  |  |  | 183901 | - | 188046 | 0.00 | 0.00 |
|  |  |  |  |  | 188046 | - | 189875 | 0.00 | 0.95 |
|  |  |  |  |  |  |  |  |  |  |
| 3 | Unknown | I | 198,866 | 202,777 | 198648 | - | 202092 | 0.31 | 0.51 |
|  |  |  |  |  |  |  |  |  |  |
| 4 | ST | I | 226,886 | 230,075 | 224761 | - | 227257 | 0.43 | 0.00 |
|  |  |  |  |  | 227257 | - | 230057 | 0.00 | 0.00 |
|  |  |  |  |  |  |  |  |  |  |
| 5 | (Ty1) | II | 265,501 | 266,187 | 258844 | - | 265486 | 0.00 | 0.00 |
|  |  |  |  |  | 265486 | - | 266540 | 1.03 | 0.00 |
|  |  |  |  |  |  |  |  |  |  |
| 6 | (LTR) | III | 148,657 | 151,244 | 139536 | - | 148655 | 0.00 | 0.00 |
|  |  |  |  |  | 148655 | - | 156945 | 1.70 | 0.84 |
|  |  |  |  |  |  |  |  |  |  |
| 7 | ST | III | 308,225 | 315,601 | 306394 | - | 310104 | 0.00 | 0.00 |
|  |  |  |  |  | 310104 | - | 313055 | 0.00 | 0.00 |
|  |  |  |  |  |  |  |  |  |  |
| 8 | ST | V | 7,823 | 10,107 | 6780 | - | 8404 | 0.00 | 0.00 |
|  |  |  |  |  | 8404 | - | 10882 | 0.00 | 0.00 |
|  |  |  |  |  |  |  |  |  |  |
| 9 | ST | VI | 15,320 | 16,784 | 8181 | - | 15963 | 0.00 | 0.00 |
|  |  |  |  |  | 15963 | - | 18000 | 0.00 | 0.00 |
|  |  |  |  |  |  |  |  |  |  |
| 10 | (Ty2) | VI | 143,952 | 144,847 | 143947 | - | 148680 | 0.00 | 0.00 |
|  |  |  |  |  |  |  |  |  |  |
| 11 | Unknown | VI | 205,005 | 205,914 | 203599 | - | 207830 | 0.77 | 0.82 |
|  |  |  |  |  |  |  |  |  |  |
| 12 | (LTR) | VII | 402,339 | 404,657 | 402842 | - | 405404 | 0.00 | 0.27 |
|  |  |  |  |  |  |  |  |  |  |
| 13 | Ty3 | VII | 707,609 | 712,241 | 706323 | - | 712714 | 0.00 | 0.00 |
|  |  |  |  |  |  |  |  |  |  |
| 14 | ST | VII | 1,069,041 | 1,076,119 |  |  | No markers |  |  |
|  |  |  |  |  |  |  |  |  |  |
| 15 | Ty4 | VIII | 85,915 | 91,934 | 84809 | - | 92110 | 0.00 | 0.00 |
|  |  |  |  |  |  |  |  |  |  |
| 16 | (LTR) | VIII | 93,293 | 94,934 | 92110 | - | 95308 | 0.34 | 0.00 |
|  |  |  |  |  |  |  |  |  |  |
| 17 | Ty3 | IX | 205,644 | 210,309 | 205763 | - | 207582 | 0.00 | 0.00 |
|  |  |  |  |  | 207582 | - | 209440 | 0.00 | 0.00 |
|  |  |  |  |  |  |  |  |  |  |
| 18 | (LTR) | IX | 300,709 | 301,477 | 300639 | - | 308351 | 0.84 | 0.90 |
|  |  |  |  |  |  |  |  |  |  |
| 19 | (LTR) | IX | 325,121 | 325,713 | 325631 | - | 331648 | 0.54 | 0.29 |
|  |  |  |  |  |  |  |  |  |  |
| 20 | ST | IX | 434,376 | 437,036 | 434310 | - | 439767 | 0.00 | 0.32 |
|  |  |  |  |  |  |  |  |  |  |
| 21 | ST | X | 21,255 | 25,375 | 21723 | - | 23668 | 0.00 | 0.00 |
|  |  |  |  |  | 23668 | - | 27189 | 0.00 | 0.00 |
|  |  |  |  |  |  |  |  |  |  |
| 22 | Ty4 | X | 197,837 | 203,309 | 191363 | - | 201062 | 0.56 | 0.00 |
|  |  |  |  |  | 201062 | - | 203696 | 0.00 | 0.00 |
|  |  |  |  |  |  |  |  |  |  |
| 23 | ST | XIV | 777,020 | 781,260 | 774474 | - | 776526 | 0.00 | 0.00 |
|  |  |  |  |  | 776526 | - | 781233 | 0.23 | 0.37 |
|  |  |  |  |  |  |  |  |  |  |
| 24 | ST | XV | 1,118 | 11,029 | 193 | - | 3719 | 0.00 | 0.49 |
|  |  |  |  |  | 3719 | - | 7779 | 0.00 | 0.43 |
|  |  |  |  |  | 7779 | - | 15878 | 0.13 | 0.00 |
| 25 | ST | XV | 1,073,295 | 1,074,365 |  |  | No Markers |  |  |
|  |  |  |  |  |  |  |  |  |  |
| 26 | ST | XVI | 16,737 | 17,512 | 15489 | - | 18930 | 0.31 | 1.01 |
|  |  |  |  |  |  |  |  |  |  |
| 27 | Ty4 | XVI | 437,284 | 442,703 | 435600 | - | 443789 | 0.00 | 0.21 |
|  |  |  |  |  | 443789 | - | 445963 | 0.00 | 0.00 |

1. For LSP descriptions, see Table S2.
2. Coordinates indicate the position of the two flanking markers for each region where recombination events were identified.
3. The number of identified reciprocal crossovers (CO) and non-crossover gene conversion (NCOGC) events in 120 tetrads for each region is divided with the expected number of events based on the size of the region (1=expected number of event). The genome average is 6.5 per Mb per meiosis for COs and 4.7 per Mb per meiosis for NCOGCs.
