# Supplementary material for: Genomic Structure of and Genome-Wide Recombination in the Saccharomyces cerevisiae S288C Progenitor Isolate EM93
Source: PLoS One. 2011 Sep 26;6(9):e25211. doi: 10.1371/journal.pone.0025211 (PMC3180460; doi:10.1371/journal.pone.0025211)
Supplement: Table S9 — Non-Crossover Gene Conversion frequency and tetrad segregation. (DOC) [file pone.0025211.s017.doc]

**TABLE S9**

**Non-Crossover Gene Conversion frequency and tetrad segregation**

| NCOGC Rank | Chr. # | Coordinates of NCOGC SNP | Total # of NCOGCs | Coordinates of markers flanking NCOGC SNP | Total # of tetrads | # of PD tetrads | # of TT tetrads | # of NPD tetrads |
| --- | --- | --- | --- | --- | --- | --- | --- | --- |
| 1 | 1 | 207154 | 24 | 202364-224761 | 120 | 116 | 4 | 0 |
| 2 | 16 | 727489 | 18 | 726624-730555 | 120 | 116 | 3 | 1 |
| 3 | 10 | 219325 | 13 | 218051-220352 | 120 | 119 | 1 | 0 |
| 4 | 6 | 35896 | 12 | 32870-36516 | 120 | 118 | 2 | 0 |
| 5 | 9 | 55057 | 12 | 48869-65509 | 120 | 99 | 21 | 0 |
| 6 | 10 | 705481 | 12 | 698817-705760 | 120 | 107 | 13 | 0 |
| 7 | 15 | 987472 | 12 | 985916-989604 | 120 | 112 | 8 | 0 |
| 8 | 8 | 506124 | 11 | 469077-522577 | 120 | 78 | 41 | 1 |
| 9 | 11 | 492956 | 11 | 491888-494246 | 120 | 119 | 1 | 0 |
| 10 | 16 | 426495 | 11 | 396191-428320 | 120 | 97 | 23 | 0 |
| 11 | 6 | 226159 | 10 | 207830-228481 | 120 | 72 | 47 | 1 |
| 12 | 6 | 245474 | 10 | 234568-258527 | 120 | 101 | 19 | 0 |
| 13 | 9 | 166164 | 10 | 164868-167620 | 120 | 119 | 1 | 0 |
| 14 | 15 | 71614 | 10 | 68376-74107 | 120 | 115 | 4 | 1 |
| 15 | 2 | 46708 | 9 | 40974-118939 | 120 | 107 | 13 | 0 |
| 16 | 7 | 167431 | 9 | 165776-171277 | 120 | 119 | 1 | 0 |
| 17 | 8 | 123594 | 9 | 121713-125757 | 120 | 114 | 6 | 0 |
| 18 | 9 | 266704 | 9 | 260006-268168 | 120 | 114 | 6 | 0 |
| 19 | 13 | 168927 | 9 | 168076-174961 | 120 | 117 | 3 | 0 |
| 20 | 15 | 340372 | 9 | 338394-340732 | 120 | 120 | 0 | 0 |
| 21 | 15 | 830761 | 9 | 801267-831037 | 120 | 82 | 38 | 0 |
| 22 | 3 | 246792 | 8 | 244165-249279 | 120 | 111 | 9 | 0 |
| 23 | 4 | 316797 | 8 | 308239-316841 | 120 | 109 | 11 | 0 |
| 24 | 7 | 293241 | 8 | 286692-293253 | 120 | 119 | 1 | 0 |
| 25 | 8 | 141666 | 8 | 139384-142275 | 120 | 117 | 3 | 0 |
| 26 | 9 | 47640 | 8 | 45971-48869 | 120 | 117 | 3 | 0 |
| 27 | 9 | 96848 | 8 | 91092-99744 | 120 | 112 | 8 | 0 |
| 28 | 11 | 580437 | 8 | 578505-580474 | 120 | 120 | 0 | 0 |
| 29 | 11 | 630662 | 8 | 628758-631654 | 120 | 112 | 8 | 0 |
| 30 | 13 | 79932 | 8 | 64448-79936 | 120 | 107 | 13 | 0 |
| 31 | 14 | 77011 | 8 | 72709-77160 | 120 | 117 | 3 | 0 |
| 32 | 14 | 180729 | 8 | 161254-184130 | 120 | 109 | 11 | 0 |
| 33 | 15 | 177004 | 8 | 176456-177722 | 120 | 119 | 1 | 0 |
| 34 | 15 | 602243 | 8 | 601743-603028 | 120 | 115 | 5 | 0 |
| 35 | 16 | 47122 | 8 | 44104-52999 | 120 | 111 | 9 | 0 |
| 36 | 1 | 196098 | 7 | 190132-198648 | 120 | 86 | 34 | 0 |
| 37 | 2 | 193537 | 7 | 191518-198087 | 120 | 111 | 9 | 0 |
| 38 | 2 | 290464 | 7 | 280672-302383 | 120 | 98 | 22 | 0 |
| 39 | 5 | 26041 | 7 | 24894-26667 | 120 | 118 | 2 | 0 |
| 40 | 5 | 43058 | 7 | 39169-46317 | 120 | 112 | 8 | 0 |
| 41 | 7 | 407444 | 7 | 405424-408707 | 120 | 119 | 1 | 0 |
| 42 | 7 | 731074 | 7 | 730219-734615 | 120 | 120 | 0 | 0 |
| 43 | 8 | 121713 | 7 | 105511-123594 | 120 | 111 | 9 | 0 |
| 44 | 8 | 289016 | 7 | 286229-290523 | 120 | 118 | 2 | 0 |
| 45 | 9 | 268168 | 7 | 266704-272654 | 120 | 117 | 3 | 0 |
| 46 | 9 | 375196 | 7 | 362705-378374 | 120 | 111 | 9 | 0 |
| 47 | 10 | 188446 | 7 | 184143-191363 | 120 | 115 | 5 | 0 |
| 48 | 10 | 316435 | 7 | 313728-319675 | 120 | 111 | 9 | 0 |
| 49 | 11 | 166676 | 7 | 165239-167774 | 120 | 114 | 6 | 0 |
| 50 | 12 | 40540 | 7 | 35464-42230 | 120 | 107 | 13 | 0 |

For markers flanking the NCOGC SNP, PD = Parental Di-type; TT = Tetratype; NPD = Non-Parental Di-type.
